# A Serum Resistant Polymer with Exceptional Endosomal Escape and mRNA Delivery Efficacy for CRISPR Gene Therapy

**DOI:** 10.1002/advs.202413006

**Published:** 2025-02-08

**Authors:** Jia Lv, Qianqian Fan, Yirou Zhang, Xujiao Zhou, Panting Yu, Xin Yu, Changchang Xin, Jiaxu Hong, Yiyun Cheng

**Affiliations:** ^1^ Shanghai Frontiers Science Center of Genome Editing and Cell Therapy Shanghai Key Laboratory of Regulatory Biology School of Life Sciences East China Normal University Shanghai 200241 China; ^2^ Department of General Surgery Center for Metabolism Research The Fourth Affiliated Hospital of Zhejiang University School of Medicine International School of Medicine International Institutes of Medicine Zhejiang University Yiwu 322000 China; ^3^ Department of Ophthalmology and Vision Science Eye, Ear, Nose, and Throat Hospital Fudan University Shanghai 200030 China; ^4^ Department of Ophthalmology Eye & ENT Hospital State Key Laboratory of Molecular Engineering of Polymers Fudan University Shanghai 200031 China; ^5^ NHC Key laboratory of Myopia and Related Eye Diseases Shanghai 200031 China; ^6^ Shanghai Engineering Research Center of Synthetic Immunology Shanghai 200032 China; ^7^ Department of Ophthalmology Children's Hospital of Fudan University National Pediatric Medical Center of China Shanghai 200031 China

**Keywords:** CRISPR gene therapy, fluoropolymer, mRNA delivery, nanoparticle, polymer

## Abstract

Nanoparticle‐based mRNA delivery offers a versatile platform for innovative therapies. However, most of the current delivery systems are limited by poor serum tolerance, suboptimal endosomal escape and mRNA delivery efficacy. Herein, a highly efficient mRNA‐delivering material is identified from a library of fluoropolymers. The lead material FD17 shows exceptional serum stability and endosomal escape, enabling efficient mRNA delivery into various cell types, surpassing commercial mRNA delivery reagents such as Lipofectamine 3000. The formed mRNA nanoparticles adsorb abundant serum albumin on the surface, which facilitates cellular uptake via scavenger receptor‐mediated endocytosis. FD17 enables the delivery of mRNAs encoding CRE, Cas9, and base editor hyCBE for efficient genome editing. The material mediates CRISPR/Cas9 gene therapy via intraocular injection effectively down‐regulates vascular endothelial growth factor A in retinal pigment epithelial cells of mice, yielding promising therapeutic responses against laser‐induced choroidal neovascularization. The discovered material in this study shows great promise for the development of mRNA therapeutics to combat a wide range of diseases.

## Graphical abstract

A fluoropolymer, FD17, identified in a polymer library, exhibits exceptional serum stability and endosomal escape, enabling efficient mRNA delivery. FD17 facilitates the intracellular delivery of mRNAs encoding CRE, Cas9, and hyCBE for genome editing. The FD17/Cas9 mRNA/VEGFA‐targeting sgRNA complex demonstrates exceptional penetration into retinal cells following intraocular injection, yielding promising therapeutic outcomes against laser‐induced neovascularization.

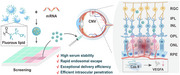

## 1 Introduction

Messenger RNA (mRNA) has emerged as a transformative therapeutic platform with the potential to address a wide array of previously incurable diseases, including cancer, genetic disorders, neurodegenerative conditions, and metabolic pathologies.^[^ ^1^ ^]^ The versatility of in vitro transcribed mRNAs allows for the production of any protein, thereby compensating for deficits caused by genetic mutations and enabling the expression of functional proteins. Additionally, mRNAs can encode transient antigens to elicit immune responses against tumors and pathogens or function as tools for CRISPR/Cas genome editing.^[^ ^2^, ^3^ ^]^ Unlike DNA‐based therapies, mRNA therapeutics avoid nuclear translocation, exhibit higher transfection efficiencies in non‐dividing cells, permit transient and controllable gene expression, and minimize the risk of integration into the host genome.^[^ ^4^ ^]^ However, like DNA, naked mRNA confronts significant challenges related to cellular uptake and susceptibility to enzymatic degradation. To harness the full potential of mRNA therapeutics and broaden their clinical applications, ongoing research efforts are focused on refining mRNA delivery systems suitable for in vivo applications.^[^ ^5^ ^]^

In recent years, numerous delivery systems have been developed, with lipid nanoparticles (LNPs) gaining prominence as effective carriers for mRNA vaccines, such as those against SARS‐CoV‐2, which have demonstrated profound efficacy in preventing COVID‐19 infections.^[^ ^6^, ^7^, ^8^ ^]^ LNPs have been extensively investigated as mRNA delivery vehicles and have shown promising results in both preclinical and clinical studies.^[^ ^9^, ^10^ ^]^ Nonetheless, LNPs face several limitations, including inadequate endosomal escape, stringent storage and transportation requirements, and inflammatory and toxic side effects.^[^ ^9^, ^11^, ^12^, ^13^ ^]^ As an alternative, polymers such as polyethyleneimine (PEI), poly(lactic‐co‐glycolic acid) (PLGA), poly(amino acids), and poly(β‐amino ester) offer structural flexibility and stability for mRNA delivery.^[^ ^9^, ^14^, ^15^, ^16^, ^17^ ^]^ These polymers are also cost‐effective and have straightforward synthesis processes. Cationic polymers typically complex with mRNA through ionic interactions, which can be disrupted by salts and serum proteins.^[^ ^18^ ^]^ To enhance complex stability, excessive amounts of polymer are often used, which can increase cytotoxicity and impede mRNA release within cells. Moreover, the strong positive charge of these complexes can lead to interactions with serum proteins, destabilizing them and compromising delivery efficacy. Therefore, the development of polymeric carriers that can effectively bind mRNA to form stable complexes under physiological conditions at low charge ratios, while also ensuring efficient endosome escape and intracellular release, is critical. The incorporation of hydrophobic lipids into polymers has been shown to improve complex stability, allowing for high transfection efficiency at low nitrogen to phosphorus (N/P) ratios.^[^ ^19^, ^20^ ^]^ However, these complexes are sensitive to phospholipid interactions on the cell membrane, which can lead to premature cargo release and reduced delivery efficiency. Fluoropolymers, as a distinct class of amphiphilic polymers, exhibit remarkable self‐assembly properties, enabling the formation of stable complexes with nucleic acids at very low N/P ratios.^[^ ^21^, ^22^ ^]^ These complexes resist protein interference and avoid blending with phospholipids on cell membrane due to the hydrophobic and lipophobic characteristics of fluoroalkyl chains, maintaining high stability and enabling efficient delivery even in serum‐containing media.^[^ ^23^, ^24^, ^25^ ^]^ Based on these rationales, we pursued a strategic approach to integrate fluoroalkyl chains into cationic polymers for the screening of highly efficient mRNA delivery carriers. By carefully modulating the degree of fluorination, we can finely control the surface charge density of these polymers, tailoring their interactions with mRNA molecules.

**Figure 1:**
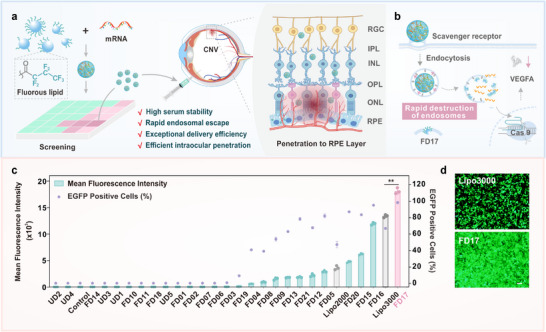
Screening and evaluation of fluoropolymers for intracellular mRNA delivery and gene editing in the treatment of age‐related macular degeneration (AMD). a) Identification of optimal fluoropolymer for intracellular mRNA delivery and choroidal neovascularization (CNV) therapy within AMD. A library of fluoropolymers was screened to identify FD17 as the lead candidate, demonstrating outstanding performance in the intraocular delivery of Cas9 mRNA and sgRNA targeting VEGFA specifically. Following delivery, the complex efficiently penetrates ocular tissues to reach the retinal pigment epithelial (ARPE‐19E) cell layer, where it effectively knocks out the VEGFA gene, reducing its expression and providing a therapeutic intervention against CNV. b) FD17 enhances intracellular delivery of Cas9 mRNA and sgRNA, resulting in gene editing that specifically downregulates VEGFA expression in ARPE‐19E cells. c) EGFP mRNA delivery efficiency of fluoropolymers in 143B cells at optimal N/P ratios. Statistically significant differences were determined between the FD17 and Lipo3000 groups using Student's *t*‐test, one‐tailed, ^**^ *p* < 0.01. d) Fluorescence images of 143B cells treated with Lipo 3000/EGFP mRNA and FD17/EGFP mRNA complexes. The scale bar is 100 µm. The concentration of EGFP mRNA was 0.8 µg mL^−1^, and the N/P ratio of the FD17 to mRNA was 2.5.

In this study, we identified FD17, an efficient mRNA delivery material, from a library of fluorinated polymers (**Figure** **1**a). The self‐assembly property of FD17 inherently facilitates the formation of highly stable mRNA nanoparticles under physiological conditions. Notably, the adsorption of serum albumin onto the nanoparticle surface not only maintains complex stability but also enhances cellular uptake through scavenger receptor‐mediated endocytosis (Figure 1b). FD17 exhibits remarkable endosomal escape capabilities, which is a key factor in its superior mRNA delivery efficiency across various cell lines, outperforming the commercial reagents such as Lipofectamine 3000 (Lipo 3000). FD17 effectively delivers mRNAs encoding gene editing tools of different sizes, including CRE, Cas9, and the base editor hyCBE, enabling precise and efficient genome editing within living cells. Furthermore, intravitreal injection of complexes formed by FD17 and Cas9 mRNA, along with sgRNA targeting vascular endothelial growth factor A (VEGFA) in mice, significantly reduced VEGFA expression in retinal pigment epithelial cells and inhibited laser‐induced choroidal neovascularization. These findings underscore the potential of FD17 in advancing the development of mRNA‐based therapeutics for a broad range of diseases.

## 2 Results and Discussion

### 2.1 Screening of Fluorinated Polymers for Intracellular mRNA Delivery

**Figure 2:**
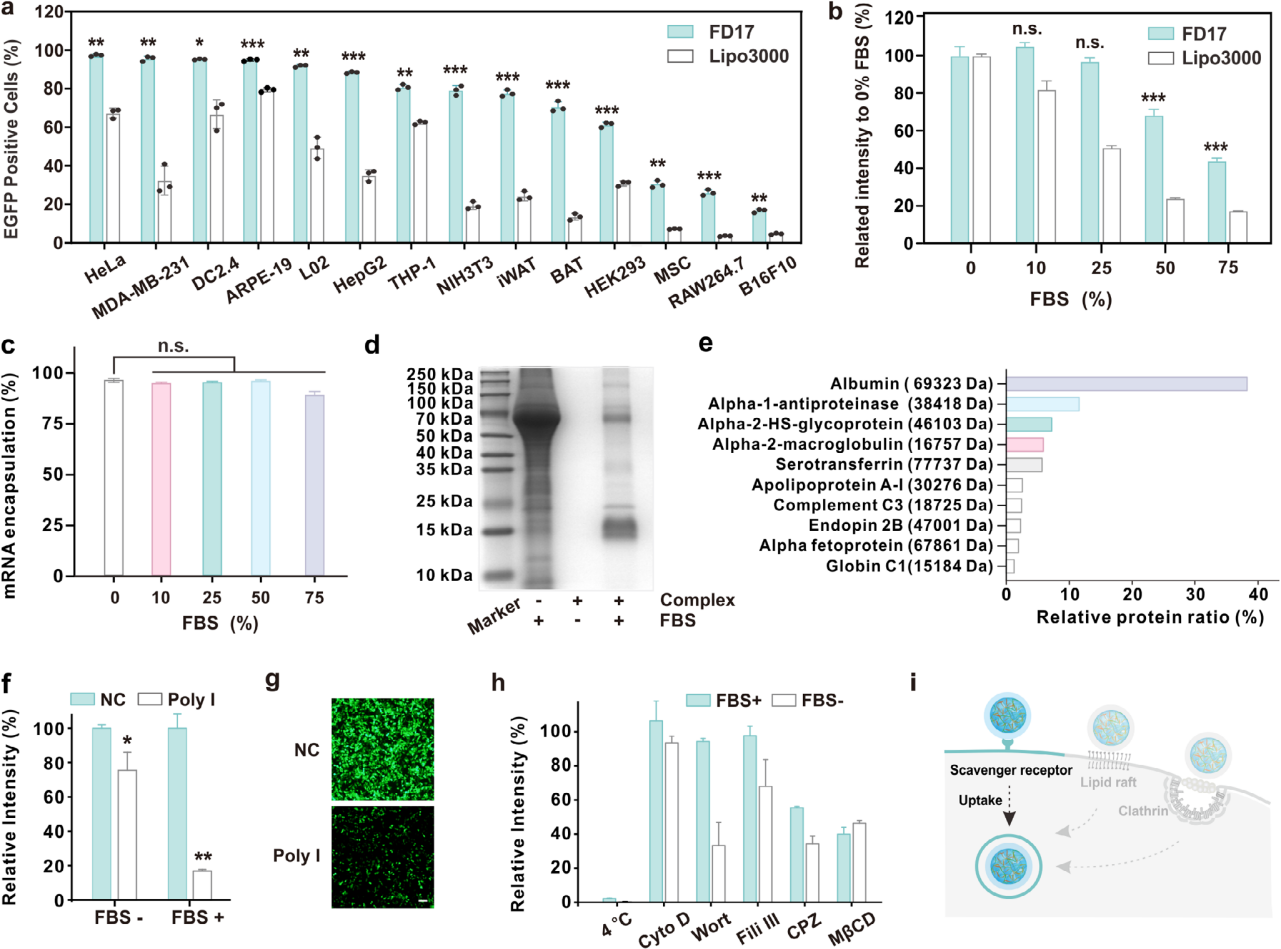
Cytosolic mRNA delivery behaviors of the FD17/mRNA complexes. a) Comparative analysis of EGFP mRNA delivery efficiency between FD17 and Lipo 3000 on various cell lines. b) mRNA delivery efficiency on 143B cells and c) mRNA encapsulation ratios of FD17 in the presence of different percentages of FBS. d) SDS‐PAGE and e) LC‐MS analysis of the protein corona on FD17/mRNA complexes in 10% FBS‐containing media. f) Relative fluorescence intensity of 143B cells treated with FD17/EGFP mRNA complexes after pretreatment with Poly I for 2 h. g) Fluorescence images of 143B cells treated with FD17/EGFP mRNA complexes in 10% FBS‐containing medium after pretreatment with Poly I for 2 h. The scale bar is 100 µm. h) Relative fluorescence intensity of 143B cells treated with FD17/EGFP mRNA complexes following pretreatment with various inhibitors. The fluorescence intensity of the cells without inhibitor pretreatment (NC) was set as 100%. i) Cell internalization pathways of the FD17/mRNA complexes. The concentration of EGFP mRNA was 0.8 µg mL^−1^, and the N/P ratio of the FD17 to mRNA complex was 2.5. Statistically significant differences were assessed using a one‐tailed Student's *t*‐test between: the FD17 and Lipo3000 groups for the data in (a); for the data in (b) and (c), within each material‐treated group, the groups with varying percentages of FBS were compared separately to the 0% FBS group within the same material‐treated group; the NC and Poly I pretreated groups for the data in (f). ^n.s.^ *p* ≥ 0.05, ^*^ *p* < 0.05, ^**^ *p* < 0.01, and ^***^ *p* < 0.001.

We synthesized a series of fluorinated poly(amidoamine) (PAMAM) dendrimers (FDn) by reacting generations 1 to 5 of PAMAM dendrimers with heptafluorobutyric anhydride at different feeding molar ratios (Figure S1, Supporting Information). This resulted in a library of 21 fluorinated dendrimers. To assess their mRNA delivery capabilities, we used mRNA expressing enhanced green fluorescent protein (EGFP) as the cargo molecule. We first evaluated the performances of the materials at varying N/P ratios, maintaining a constant mRNA concentration of 0.8 µg mL^−1^ (Figures S2–S6, Supporting Information). The mRNA delivery efficiency for each polymer at its optimal N/P ratio is summarized in Figure 1c. Among the synthesized polymers, FD17, a G4 PAMAM dendrimer functionalized with 47 fluoroalkyl chains, emerged as the most effective candidate in the library, outperforming commercial reagents such as Lipofectamine 2000 (Lipo 2000) and Lipo 3000 (Figure 1d). Dynamic light scattering (DLS) analysis showed that the FD17/mRNA complexes carried a positive charge and had an average size of ≈223 nm (Figure S7, Supporting Information). FD17 not only demonstrated superior mRNA delivery in 143B cells but also exceeded the performance of Lipo 3000 across a panel of cell lines, including HeLa, MDA‐MB‐231, DC2.4, ARPE‐19E, L02, HepG2, THP‐1, NIH3T3, iWAT, BAT, HEK293, MSC, RAW264.7, and B16F10 (**Figure** **2**a). Notably, FD17 exhibited robust serum tolerance during cytosolic mRNA delivery. Cells treated with FD17/mRNA complexes maintained over 97% and 68% EGFP transfection efficacy in the presence of 25% and 50% fetal bovine serum (FBS), respectively, due to the high stability of the complexes (Figure 2b,c). In comparison, Lipo 3000 achieved only 51% and 24% mRNA delivery efficacy under the same conditions. To understand how the positively charged FD17/mRNA complexes maintain efficient delivery in the presence of serum proteins, we investigated the interactions of FD17 with FBS. Previous studies have indicated that nanoparticles like LNPs adsorb specific proteins in serum, forming protein coronas that can affect mRNA delivery efficiency.^[^ ^26^, ^27^, ^28^ ^]^ We identified albumin as the predominant protein adsorbed to the FD17/mRNA complexes by incubating them with FBS, followed by ultracentrifugation and analysis using SDS‐PAGE and liquid chromatography‐mass spectrometry (LC‐MS) (Figure 2d,e). Albumin‐coated nanoparticles have been shown to bind preferentially to glycoprotein scavenger receptors on the cell surface, facilitating internalization via receptor‐mediated endocytosis.^[^ ^29^, ^30^ ^]^ To investigate the involvement of scavenger receptors in the internalization of our complexes, we pretreated cells with the scavenger receptor inhibitor, polyinosinic acid (Poly I),^[^ ^31^ ^]^ and evaluated the cellular uptake of the complexes. The pretreatment with Poly I substantially reduced the endocytosis of the complexes in serum‐containing media (Figure 2f,g), indicating a significant role for scavenger receptor‐mediated endocytosis in the uptake of these complexes. In serum‐free conditions, the pretreatment with Poly I only slightly inhibited cellular uptake. We further investigated the endocytic pathways previously reported for cationic polymers and found that complexes formed under serum‐free conditions were primarily internalized through micropinocytosis, clathrin‐mediated endocytosis, and caveolae‐mediated endocytosis. Conversely, the internalization of nanoparticles adorned with a protein corona was associated with lipid raft‐ and clathrin‐mediated pathways, while showing a reduced dependence on micropinocytosis (Figure 2h). Overall, the FD17/mRNA complex can adsorb a protein corona predominantly composed of albumin in serum‐containing media, facilitating cellular entry via scavenger receptor‐dependent, and clathrin‐ and lipid raft‐mediated endocytic pathways (Figure 2i). Additionally, under serum‐free conditions, the complexes exhibited less dependence on scavenger receptors and primarily entered cells through a combination of pathways, including macropinocytosis, clathrin‐mediated endocytosis, lipid raft‐mediated endocytosis, and caveolae‐mediated endocytosis.

### 2.2 Endosomal Escape Study of Fluorinated Polymers

**Figure 3:**
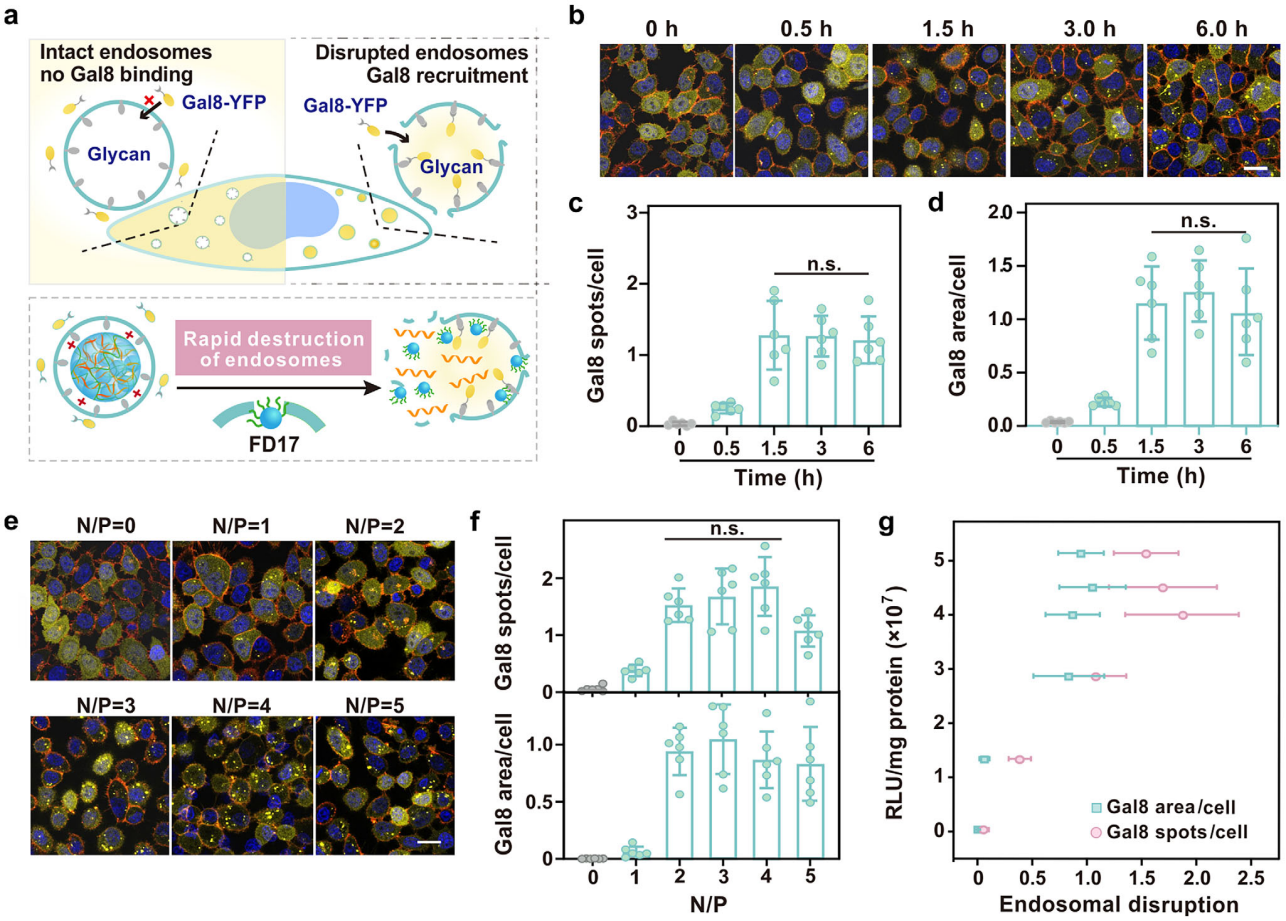
Endosomal escape behaviors of FD17. a) Schematic representation of Gal8‐YFP recruitment to disrupted endosomes and the mechanism of endosomal membrane disruption by FD17. b) Fluorescent microscopy was used to capture images of Gal8‐YFP‐HeLa cells exposed to FD17/luciferase mRNA complexes at various time points ranging from 0 to 6 h. The scale bar is 20 µm. c) Average number and d) area of Gal8‐YFP spots per cell treated with FD17/luciferase mRNA complexes over the same time course. The N/P ratio of the FD17/mRNA complex was 2.5. e) Fluorescence microscopy images showcasing Gal8‐YFP‐HeLa cells subjected to treatment with FD17/luciferase mRNA complexes at varying N/P ratios. The scale bar is 20 µm. f) Average number and area of Gal8‐YFP spots per cell treated with FD17/luciferase mRNA complexes at varying N/P ratios. The mRNA delivery experiments were conducted for 6 h. g) Correlation analysis between the level of luciferase expression and the extent of endosomal membrane disruption in cells treated with FD17/luciferase mRNA complexes. ^n.s.^ *p* > 0.05 were subjected to analysis using one‐way ANOVA, which was then followed by the Newman‐Keuls Multiple Comparison Test.

Endosomal escape is a pivotal challenge in the quest for effective intracellular mRNA delivery. To quantify the ability of FD17/mRNA complexes to overcome this barrier, we utilized a HeLa cell line stably expressing Galectin 8 (Gal8) fused with yellow fluorescent protein (YFP). Gal8, a β‐galactoside‐binding lectin, is normally distributed throughout the cytosol but quickly translocates to damaged endosomes upon membrane disruption, due to its strong affinity for galactosides in the inner leaflet of endosome membrane. This translocation is visualized as distinct fluorescent spots, signaling endosome disruption (**Figure** **3**a). Following treatment with FD17/mRNA complexes, pronounced fluorescent spots were observed within 30 min (Figure 3b–d). By 1.5 h, the fluorescent signal had saturated, indicating near‐complete endosomal escape. The swift disruption and escape are attributed to the membrane‐disturbing effect of the fluoroalkyl chains in FD17 (Figure 3a). This distinctive feature significantly enhances the bioavailability of the cargo mRNA, facilitating its rapid expression in target cells (Figure S8, Supporting Information). Importantly, the endosomal escape efficiency of FD17 is influenced by the N/P ratio, with high efficiency observed at ratios greater than 2 (Figure 3e,f). Our results reveal a direct correlation between the efficiency of endosomal escape and the efficacy of mRNA delivery (Figure 3g), underscoring the critical importance of endosomal escape in the effectiveness of mRNA delivery systems. Cells treated with Lipo3000 and UD4 showed minimal aggregation of Gal8‐YFP (Figure S9, Supporting Information), indicating significantly lower efficiency compared to the FD17/mRNA complexes. Free RNA did not significantly disrupt the endosomal membrane (Figure 3e and N/P = 0).

### 2.3 Intracellular mRNA Delivery of FD17 for Gene Editing

**Figure 4:**
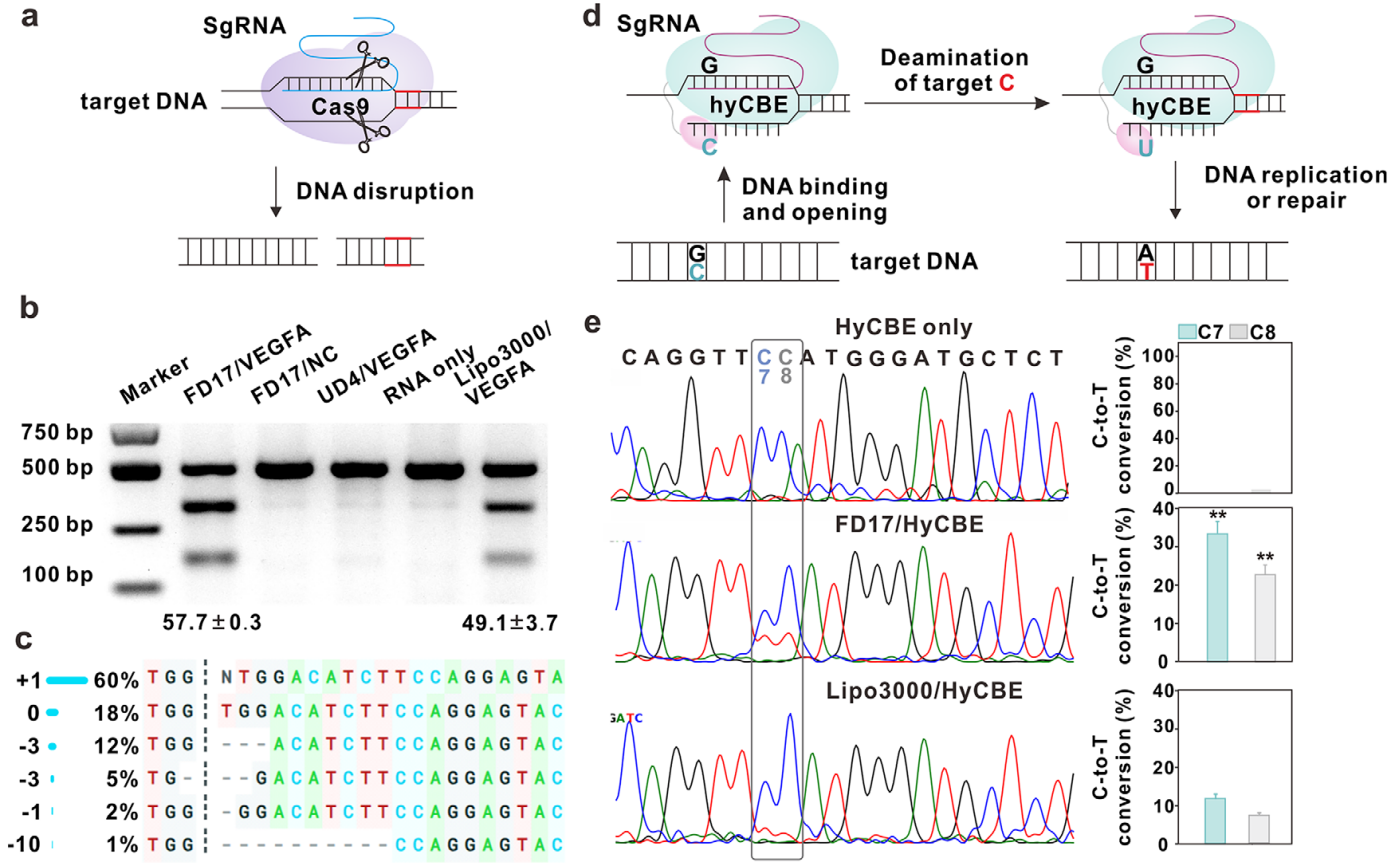
FD17‐based mRNA delivery system for gene editing in vitro and in vivo. a) Schematic illustration of Cas9 ribonucleoprotein complex for targeted gene disruption. Indel efficiency at the VEGFA locus in ARPE‐19E cells was determined by b) T7E1 assay and c) Sanger sequencing. d) Schematic illustration of hyCBE and sgRNA complexes for base editing. e) Base editing efficiency at the PCSK9 locus in Hepa 1–6 cells was determined by Sanger sequencing. Statistically significant differences were determined between the FD17/HyCBE and Lipo3000/HyCBE groups using Student's *t*‐test, one‐tailed, ^**^ *p* < 0.01.

CRISPR‐Cas9 gene editing has shown considerable promise in the treatment of a wide spectrum of genetic disorders, enabling delivery to target cells in various formats.^[^ ^32^, ^33^, ^34^ ^]^ The co‐delivery of Cas9 mRNA and guide RNA (gRNA) offers an attractive strategy to mitigate off‐target effects associated with plasmid delivery.^[^ ^35^ ^]^ Here, we assessed the capacity of FD17 to enhance the cytosolic delivery of Cas9 mRNA and sgRNA for gene editing applications (**Figure** **4**a). ARPE‐19E cells were exposed to FD17/Cas9 mRNA/sgRNA complexes targeting VEGFA. Lipo 3000 served as a positive control, while unmodified generation 4 PAMAM dendrimer (UD4)/Cas9 mRNA/sgRNA and FD17/Cas9 mRNA/scrambled sgRNA (FD17/NC) complexes were tested as negative controls. T7 endonuclease I (T7E1) digestion assays revealed that FD17/Cas9 mRNA/sgRNA complexes targeting VEGFA (FD17/VEGFA) achieved a 57.7% indel efficiency in ARPE‐19E cells, significantly outperforming Lipo 3000 (Figure 4b). Sanger sequencing confirmed base insertions and deletions at the VEGFA locus in the FD17/VEGFA group (Figure 4c). Cells treated with UD4/VEGFA and FD17/NC complexes exhibited no detectable genome editing. Encouraged by the successful delivery of the CRISPR system using FD17, we proceeded to explore the viability of base editing through the delivery of hyCBE mRNA and sgRNA, tailored for C‐to‐T substitutions at the PCSK9 locus in mouse cells (Figure 4d).^[^ ^36^ ^]^ Notably, Hepa 1–6 cells treated with FD17/HyCBE mRNA/sgPCSK9 complexes (FD17/PCSK9) achieved a remarkable base editing efficiency of 33.3%, outstripping the base editing efficiency achieved with Lipo 3000 (Figure 4e).

### 2.4 Intraocular Delivery of CRISPR‐Cas9 for the Treatment of AMD

**Figure 5:**
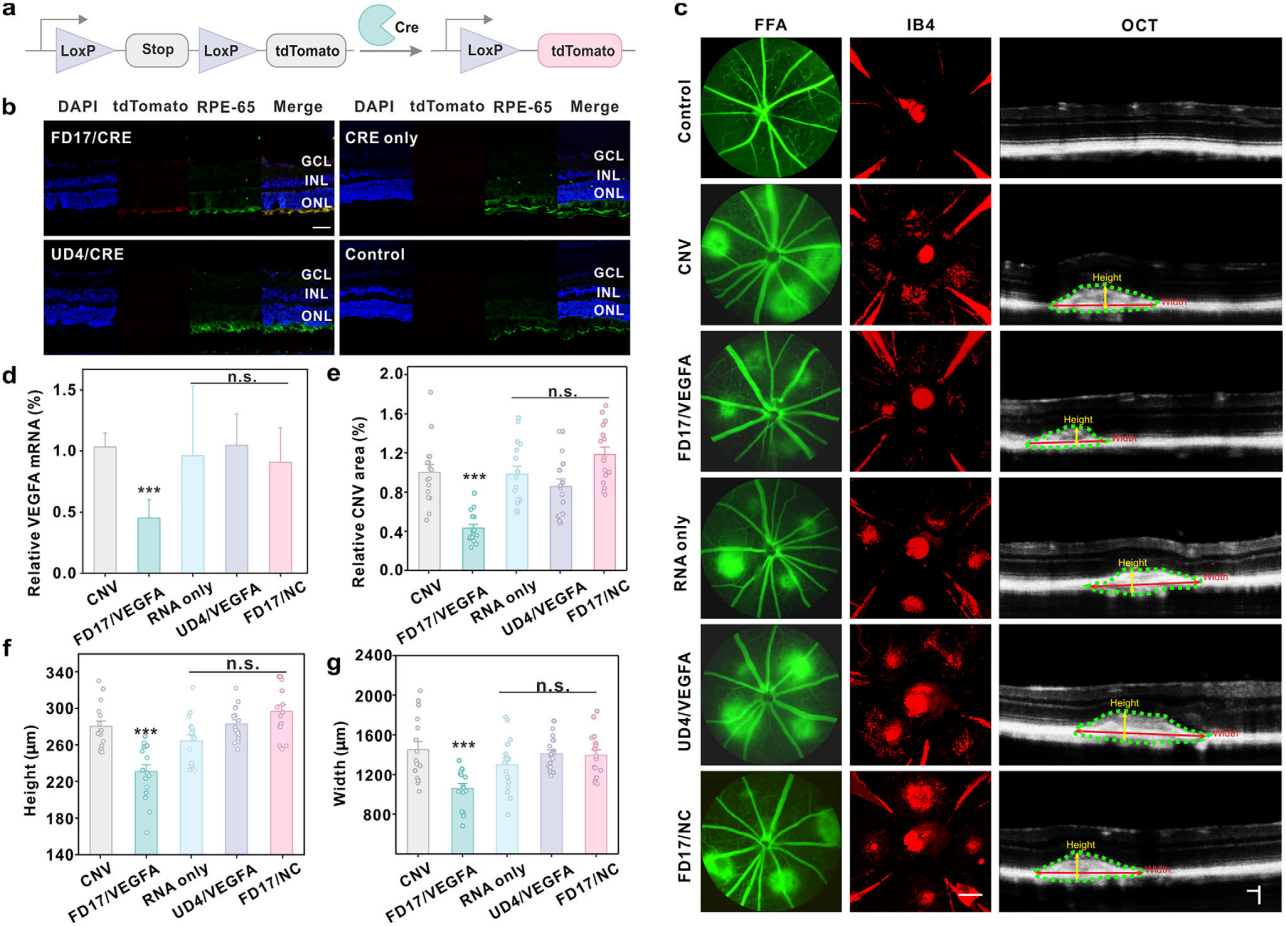
FD17‐based intraocular gene editing for the treatment of laser‐induced CNV. a) Schematic illustration of CRE‐mediated tdTomato expression in Ai14 mouse. b) Confocal microscopy image of ocular tissue layers in Ai14 mouse after intravitreal injection of FD17/CRE mRNA complexes. The scale bar is 20 µm. The untreated Ai14 mouse was used as a control. The tissue sections were stained with DAPI and ARPE‐19E‐65 to visualize the cell nuclei and the ARPE‐19E cell layer. c) Representative images of FFA, IB4, and OCT. CNV lesions were evident in experimental groups as four‐leaf clover‐shaped choroidal‐retinal flat mounts, appearing as red rounds on each leaf in the FFA images. Scale bar: 100 µm. Invading neovascularization into the ARPE‐19E was clearly visible in OCT images, and the scale bar is 200 µm. d) Relative VEGFA mRNA level in the eyeball of mice. CNV lesions were measured in 3D using image analysis software for relative CNV area e), height f), and width g). Statistically significant differences were determined between CNV and each group using Student's *t*‐test, one‐tailed, ^n.s.^ *p* ≥ 0.05 and ^***^ *p* < 0.001.

After demonstrating the excellent mRNA delivery efficiency in vitro, we further investigated its mRNA delivery potential for in vivo gene editing using Ai14 mice, which harbors a LoxP‐flanked stop cassette that disrupts tdTomato transcription. Upon CRE‐mediated excision of this cassette, tdTomato expression is induced (**Figure** **5**a). FD17/CRE mRNA complexes were administered via intravitreal injection. Five days post‐treatment, the tdTomato expression in ocular tissues was analyzed via immunohistochemical staining. As shown in Figure 5b, tdTomato expression was mainly observed in the ARPE‐19E‐65‐positive ARPE‐19E cell layer of the complexes‐treated group, suggesting effective mRNA delivery and gene editing in ARPE‐19E cells. Neovascular age‐related macular degeneration (AMD), commonly known as wet AMD, is a progressive ocular disorder characterized by the formation of abnormal blood vessels beneath the retina.^[^ ^37^, ^38^ ^]^ These vessels can leak fluid and blood, which can damage the macula, the central region of the retina essential for sharp central vision. Without intervention, wet AMD often leads to rapid and severe vision loss. AMD‐induced choroidal neovascularization primarily destroys ARPE‐19E cells, subsequently affecting Bruch's membrane, photoreceptor cell layers, and neuron cell layers, ultimately causing vision loss. The function of retinal cells depends on normal cellular structures, and abnormal angiogenesis, fluid leakage, and scarring in the macula can cause permanent central vision loss. The VEGF family of cytokines, particularly VEGFA, plays a crucial role in vasculogenesis, angiogenesis, and lymphangiogenesis, driving endothelial cell migration, proliferation, and tube formation.^[^ ^39^ ^]^ However, therapies involving repeated injections of VEGFA antagonists typically only halt disease progression and reduce the risk of vision loss, without improving visual function. These treatments also impose significant mental and financial burdens on patients and may be associated with complications.^[^ ^40^ ^]^ CRISPR‐based gene editing of the VEGFA gene, which has shown promising results in preclinical studies for suppressing choroidal neovascularization, presents a novel therapeutic strategy for enhancing the management of wet AMD. This approach has the potential to establish long‐term anti‐angiogenic treatment options for AMD.^[^ ^41^ ^]^ Based on the demonstrated efficient intracellular mRNA delivery and gene editing efficiency of FD17 in ARPE‐19E cells, we explored the feasibility of using FD17 to deliver Cas9 and sgRNA targeting VEGFA for the treatment of AMD. The pathogenic process of AMD was mimicked by laser photocoagulation, leading to choroidal neovascularization that penetrated the Bruch's membrane and ARPE‐19E layer. These choroid‐derived capillaries have high permeability and can cause apparent leakage when fluorescence is injected intraperitoneally. As a result, hyper fluorescence and leakage were observed in Fundus fluorescein angiography (FFA) images of model mice, whereas normal mice exhibited none of these symptoms (Figure 5c). The successful establishment of the AMD model was also validated through Isolectin‐IB4‐ staining and optical coherence tomography (OCT) imaging. We formulated delivery complexes (FD17/VEGFA) by combining FD17 with Cas9 mRNA and sgRNA targeting VEGFA, resulting in positively charged complexes with an average size of 200 nm. To visualize the distribution of complexes, sgRNA targeting VEGFA was labeled with Cy5.5. After intravitreal injection, the FD17/VEGFA_Cy5.5_ complexes efficiently accumulate at the ARPE‐19E layer (Figure S10, Supporting Information). Conversely, complexes formulated with the unmodified dendrimer exhibited minimal fluorescence at the ARPE‐19E layer, highlighting the beneficial effect of fluorination in ARPE‐19E accumulation. Following intravitreal injection, the delivery complex may reach the posterior segment cells through permeation across ocular cell layers and/or penetration of the blood‐retinal barrier (BRB).^[^ ^42^ ^]^ Besides the ARPE‐19E layer, tdTomato expression in mice treated with FD17/CRE mRNA complexes was also observed in the ganglion cell layer (GCL) and inner nuclear layer (INL) (Figure 5b), indicating the complex's ability to permeate ocular cell layers. To ascertain whether the delivery complex crossed the BRB, we measured the fluorescence intensity of Cy5.5 in blood samples collected subsequent to intravitreal injection of the FD17/VEGFA_Cy5.5_ complex. The detection of Cy5.5 fluorescence in the blood (Figure S11, Supporting Information) suggests that the RNA delivered via FD17 has entered the bloodstream and potentially accessed the ARPE‐19E cell layer via the bloodstream. Gene recombination mediated by Cre mRNA (Figure 5b) and the detection of Cy5.5‐labeled sgRNA (Figure S10, Supporting Information) were absent in the ocular tissue layers of mice treated with free RNA, indicating that free RNA failed to effectively penetrate ocular cell layers and be internalized by ARPE‐19E cells.

We then tested the therapeutic effect of the delivery complexes for AMD via VEGFA gene disruption. Seven days post‐intravitreal injection, the ARPE‐19E cells were isolated, and gene editing efficiency was assessed. Notably, mice treated with the FD17/VEGFA complexes achieved a 4% disruption of the VEGFA gene, outperforming the polymer or RNA alone. This reduction in VEGFA gene expression led to a substantial decrease in choroid‐derived leakage of capillary, angiogenesis, and CNV lesions in the FD17/VEGFA group (Figure 5c–g).

### 2.5 Biosafety of the FD17‐Based mRNA Delivery Complexes

**Figure 6:**
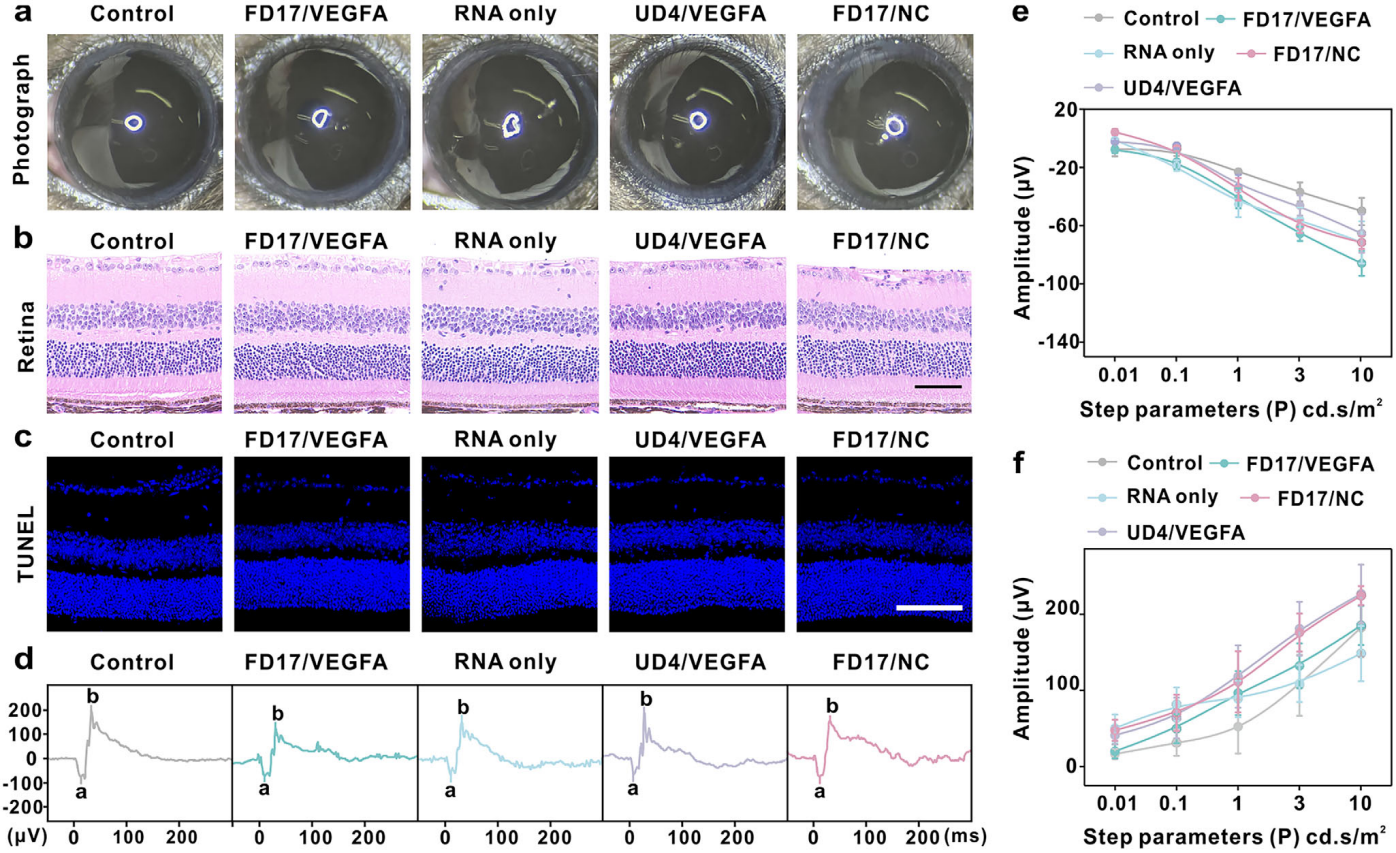
Biosafety assessment of the delivered complexes. a) Photographs of the anterior segment. b) H&E staining of the retina. Scale bar: 50 µm. c) TUNEL staining of retina. DAPI is blue, and TUNEL is red. Scale bar: 75 µm. d) Scotopic ERG responses with the stimulus applied at 10 cd^*^s m^−2^, representative a‐wave and b‐wave traces were observed in eyes from six groups. e,f) Quantitative analysis of dark‐adapted ERG responses in various luminance. The rising (e) a‐wave and f) b‐wave was elicited by an increasing flash intensity.

We finally assessed the biosafety of the delivered complexes. One week after the prescribed pharmacological regimen in normal mice, the integrity and opacity of the cornea were evaluated using anterior segment images. Clear cornea and iris regions suggest no impairment to the anterior segment across all the groups (**Figure** **6**a). Hematoxylin and eosin (H&E) and TUNEL staining of retinal tissue slices revealed intact layers without pathological abnormalities (Figure 6b,c). The absence of red fluorescence in these sections indicates minimal apoptosis in retinal cells. The ERG‐a wave primarily reflects changes in cone and rod photoreceptor potentials, while the ERG‐b wave captures the electrical properties of bipolar, Müller, and ganglion cells. Figure 6d–f depicts representative dark‐adapted ERG recordings under optimal stimulation, with quantitative analysis indicating no significant difference between the control and each group, suggesting no detrimental effects following the treatments. HE staining results revealed that no notable pathological change was observed in the ocular tissues or major organs of the mice during the treatment (Figure S12, Supporting Information).

## 3 Conclusion

In this study, we developed a library of fluoropolymers to explore their potential as efficient vectors for intracellular mRNA delivery. Our results indicate that the mRNA delivery efficiency of these polymers increased with both dendrimer generation and fluorination degree. Notably, FD17 stood out as the most effective candidate in mRNA delivery, outperforming the commercial reagents Lipo 2000 and Lipo 3000 across various cell lines. The polymer FD17 exhibits high efficacy to form stable complexes with mRNA, even in the presence of serum. In serum‐containing media, these complexes adsorb serum proteins on the surface without compromising the mRNA loading efficiency. The adsorbed albumin in turn enhances the cellular uptake of FD17/mRNA complexes through scavenger receptor‐mediated endocytosis, allowing FD17 to maintain its exceptional mRNA delivery in up to 75% serum‐containing conditions. Furthermore, FD17 demonstrates rapid endosomal escape, reaching saturation within 2 h, which facilitates mRNA expression within cells. FD17 is able to effectively bind and deliver a wide range of cargo mRNA, including CRE, Cas9, and HyCBE, enabling precise gene editing applications. In an ocular setting, FD17 shows exceptional penetration into retinal pigment epithelium cells following intravitreal injection of a complex containing FD17, Cas9 mRNA, and VEGFA‐targeting sgRNA in a mouse model of laser‐induced choroidal neovascularization. This efficient delivery led to efficient disruption of the VEGFA gene, significantly reducing choroidal capillary leakage, angiogenesis, and CNV lesions, suggesting its potential as an efficient delivery system for the treatment of age‐related macular degeneration. This study broadens the application scope of fluoropolymers, as well as offers novel perspectives on the design of mRNA delivery vectors, driving progress in gene therapy and related fields.

## Conflict of Interest

The authors declare no conflict of interest.

## Supporting information



Supporting Information

## Data Availability

The data that support the findings of this study are available from the corresponding author upon reasonable request.
