# Supplementary material for: A Serum Resistant Polymer with Exceptional Endosomal Escape and mRNA Delivery Efficacy for CRISPR Gene Therapy
Source: Adv Sci (Weinh). 2025 Feb 8;12(13):2413006. doi: 10.1002/advs.202413006 (PMC11967772; doi:10.1002/advs.202413006)
Supplement: Supplementary file 1 — Supporting Information [file ADVS-12-2413006-s001.docx]

Supporting Information

A Serum Resistant Polymer with Exceptional Endosomal Escape and mRNA Delivery Efficacy for CRISPR Gene Therapy

Jia Lv^1†^, Qianqian Fan^1,2†^, Yirou Zhang^3†^, Xujiao Zhou^3^, Panting Yu^1^, Xin Yu^1^, Changchang Xin^3^, Jiaxu Hong^3,4,5,6,7,^*, Yiyun Cheng^1,^*

^†^These authors contributed equally to this work.

^1^Shanghai Frontiers Science Center of Genome Editing and Cell Therapy, Shanghai Key Laboratory of Regulatory Biology, School of Life Sciences, East China Normal University, Shanghai, 200241, China

^2^Department of General Surgery, Center for Metabolism Research, the Fourth Affiliated Hospital of School of Medicine, and International School of Medicine, International Institutes of Medicine, Zhejiang University, Yiwu, 322000, China

^3^Department of Ophthalmology and Vision Science, Shanghai Eye, Ear, Nose and Throat Hospital, Fudan University, Shanghai, 200030, China

^4^Department of Ophthalmology, Eye & ENT Hospital, State Key Laboratory of Molecular Engineering of Polymers, Fudan University, Shanghai 200031, China.

^5^NHC Key laboratory of Myopia and Related Eye Diseases Shanghai, 200031, China.

^6^Shanghai Engineering Research Center of Synthetic Immunology, Shanghai, 200032, China.

^7^Department of Ophthalmology, Children's Hospital of Fudan University, National Pediatric Medical Center of China, Shanghai, 200031, China.

*Corresponding author. Email: Jiaxu.hong@fdeent.org (J. Hong); yycheng@mail.ustc.edu.cn (Y. Cheng).

Experimental Section

**Materials:** Ethylenediamine-cored and amine-terminated PAMAM dendrimers of generations G1, G2, G3, G4, and G5 were purchased from Dendritech (Midland, USA). Heptafluorobutyric anhydride, wortmannin, polyinosinic acid, and genistein were obtained from Sigma-Aldrich (St. Louis, USA). Filipin III was sourced from Yuanye Bio (Shanghai, China). Triethylamine, ninhydrin hydrate, methyl-β-cyclodextrin, and chlorpromazine were purchased from Macklin (Shanghai, China). Lipofectamine 2000 (Lipo2000) and Lipofectamine 3000 (Lipo3000) were acquired from Thermo Fisher Scientific, Inc. (Waltham, USA). Luciferase assay system and cell lysis buffer were obtained from Promega (Wisconsin, USA). Hoechst 33342 was acquired from Beyotime Biotechnology (Shanghai, China). Firefly Luciferase mRNA (ARCA, 5mCTP, ψUTP) (Catalog No. R1005), ARCA EGFP mRNA (5-moUTP) (Catalog No. R1007), and Cas9 mRNA (5-moUTP) (Catalog No. R1015) were sourced from APExBIO (Houston, USA). HyCBE mRNA and CRE mRNA were gifts from Prof. Dali Li (East China Normal University). ARPE-19 (CL0026) was purchased from Procell Life Science&Technology Co.,Ltd (Wuhan, China)

**Synthesis and characterization of fluoropolymers:** Heptafluorobutyric anhydride and dendrimers were dissolved in methanol and mixed at different molar ratios (Table S1), then added with triethylamine (the molar ratio of triethylamine to heptafluorobutyric anhydride was 1.5:1) to neutralize the yielding acids. The mixture solution was stirred at room temperature for 48 h. After that, for fluorinated G1 PAMAM dendrimers, the reaction solution was concentrated and added with excess diethyl ether to obtain the solid products. The products were further dissolved in methanol and reprecipitated in diethyl ether twice, then dried under vacuum to obtain the fluoropolymers as light-yellow gels. For fluorinated G2 to G5 PAMAM dendrimers, the products were dialyzed against 0.6 M hydrochloric acid solution and distilled water, then lyophilized to obtain the fluorinated dendrimers as white gels. The average number of fluoroalkanes on each polymer was tested by a well-established ninhydrin assay^1^. The fluoroalkanes modification number of the top four materials, namely FD17, FD16, and FD15 were also verified by fluorine element analysis (Shanghai Institute of Organic Chemistry, China), and the average number of fluoroalkanes modified on each polymer was calculated according to the Eq. (1)

$x=\frac{Mw\left( UD \right)\times wt\%}{7\times Mw\left( F \right)-Mw\left( CF \right)\times wt\%}$ （1）

x represents the number of fluoroalkanes attached to the polymer surface. Mw (UD) denotes the relative molecular weight of unmodified dendrimers, while wt% indicates the mass percentage of fluorine. Mw (F) represents the relative atomic weight of a fluorine atom, and Mw (CF) signifies the relative molecular weight of the fluorinated alkyl chain grafted onto the polymer surface.

The feeding molar ratios of heptafluorobutyric anhydride to the dendrimers and the average numbers of fluoroalkanes conjugated on each dendrimer are shown in Table S1. The structure of FD17 was also verified by MALDI-TOF mass spectrometry (Figure S1). Due to the abundance of amino groups on the dendrimer surface, the direct mixing of heptafluorobutyric anhydride with the dendrimer does not allow for precise control over the number of fluoroalkyl chains attached to each macromolecule. Therefore, the resulting material is likely a mixture of dendrimers with varying numbers of fluoroalkyl chains. The conjugated number obtained from the ninhydrin assay, fluorine elemental analysis, and MALDI-TOF represent an average attachment number of fluoroalkyl chains per dendrimer surface.

**Cell culture:** HeLa (human cervical carcinoma cell, ATCC), MDA-MB-231 (human breast carcinoma cell, ATCC), 143B (human osteosarcoma cell, ATCC), iWAT (mouse inguinal white adipocyte cell, a gift from Prof. Qiurong Ding working in the Chinese Academy of Sciences), BAT (mouse brown adipose tissue cell, a gift from Prof. Dongning Pan working in Fudan University), NIH3T3 (mouse embryo fibroblast cell, ATCC), HepG2 (human hepatoma cell, ATCC), RAW 264.7 (mouse leukemic monocyte macrophage cell, ATCC), HEK293 (human embryonic kidney cell, ATCC), MSC (mesenchymal stem cells, ATCC), B16F10 (mouse melanoma cells, ATCC), ARPE-19 (human RPE cell line, Procell Life Science&Technology Co.,Ltd ), and LX-2 (human hepatic stellate cell, ATCC) were cultured in Dulbecco's Modified Eagle Medium (DMEM, GIBCO) containing penicillin sulphate (100 units/mL), streptomycin (100 mg/mL) and 10% heat-inactived fetal bovine serum (FBS, GIBCO). DC2.4 (a murine bone marrow-derived [dendritic cell line](https://www.sciencedirect.com/topics/immunology-and-microbiology/dendritic-cell-line), ATCC) and L02 (a human hepatocyte cell line, ATCC) were cultured in Roswell Park Memorial Institute 1640 medium (RPMI-1640, GIBCO) containing penicillin sulphate (100 units/mL), streptomycin (100 mg/mL) and 10% FBS. THP-1 (human acute monocytic leukemia cell, ATCC) were maintained in RPMI-1640 containing penicillin sulphate (100 units/mL), streptomycin (100 mg/mL), 2-mercaptoethanol (0.05 mM). To differentiate into a macrophage phenotype, 200 nM PMA was added to the culture medium of THP-1 cells and incubated for 24 hours, then the cells were refreshed with culture medium without PMA, and incubated for 48 hours before further use.

**Preparation and characterization of the polymer/mRNA complexes:** mRNA was mixed with the fluoropolymers at different N/P ratios in 15 μL RNase free water and incubated at room temperature for 15 min, then 35 μL RNase free water or FBS-free cell culture medium was added to the above solution and incubated for 15 min. The complexes were diluted with RNase free water or cell culture medium (with 0%, 10%, 20%, 50%, or 75% FBS) before characterization or mRNA delivery experiments. The size and zeta potentials of the complexes were characterized by Zetasizer (Malvern, UK). The morphologies of the complexes were observed by cryogenic transmission electron microscope (Thermo Fisher, USA). The RNA encapsulation ratios of the polymers were tested by Ribogreen assay. In brief, the LNP solution was lysed with Triton-X-100, and the total RNA content within the solution was quantified through RiboGreen detection. Subsequently, an equivalent volume of LNP solution was detected by RiboGreen without Triton-X-100 treatment. The RNA encapsulation ratios were by dividing the difference in RNA quantity before and after lysis by the total RNA quantity in the solution post-lysis.

**In vitro mRNA delivery:** The cells were cultured in 24-well plates for 12-24 h until 80-90% confluence, then added with the polymer/protein complexes. After 6 h incubation, the cells were added with 500 μL [cell culture medium](javascript:;) containing 10 % FBS and further incubated for 18 h. The EGFP expression level was observed by a fluorescent microscopy (Olympus, Japan) and quantitatively analyzed by flow cytometry (BD, USA). The expression of luciferase was assessed following the guidelines provided by the manufacturer (Promega), with the luciferase activity levels adjusted based on protein concentrations (measured as relative luciferase light units per milligram of protein). Commercial reagents Lipofectamine 2000 and Lipofectamine 3000 were tested as positive controls, n=3.

**Protein corona identification:** The polymer/mRNA complexes were incubated in PBS supplemented with 10% FBS for 30 min, followed by centrifugation at 4°C and 10000 rpm for 20 min to separate the components. The supernatant was discarded, and the precipitate was resuspended in 50 μL of RNase-free water. For SDS-PAGE analysis, the resuspended precipitate was heated to 100°C for 10 min, mixed with loading buffer, and loaded onto a 10% acrylamide gel for electrophoresis at 120 V for 30 min. The proteins within the gel were visualized using Coomassie brilliant blue staining. For protein identification, the sample was subjected to LC-MS/MS analysis by Shanghai Bio-Pioneer Biotechnology, and the data were analyzed using MaxQuant 2.0.1.0.

**Gene editing assay:** For VEGFA gene editing, RPE cells were seeded in 24-well plates and incubated for 12-24 h to achieve 40% confluence. A mixture of 0.1 μg Cas9 mRNA and 0.05 μg sgRNA (CUCCUGGAAGAUGUCCACCA) targeting VEGFA was formulated and complexed with FD17 at an N/P ratio of 4. To this mixture, 15 μL of RNase-free water was added, followed by incubation at room temperature for 15 min. Subsequently, 35 μL of FBS-free cell culture medium was incorporated and incubated for another 15 min. The complexes were then diluted with 200 μL of FBS-free medium before replacing the media in the 24-well plates with the complex mixture. After 6 h of incubation, 500 μL of cell culture medium containing 10% FBS was added to the cells. Following 72 h of mRNA delivery, the editing efficiency was assessed using both T7E1 (T7 Endonuclease I) assay and Sanger sequencing. Genomic DNA was extracted from the treated cells using the FastPure Cell/Tissue DNA Isolation Mini Kit (DC102, Vazyme) as per standard protocols. The extracted DNA was then amplified with two pairs of specific PCR primers (F: CAGGCCATGTCCCTTGGAAC; R: CCAAAGATGCCCACCTGCAT) using Phanta Max Master Mix (P515, Vazyme) to generate templates for Sanger sequencing. For the T7E1 assay, the PCR products underwent a denature/annealing process in a thermocycler, and 20 U of T7E1 (E3321S, Biolabs) was added. Following a 40-min incubation at 37°C, the digestion products were analyzed by 2% (w/v) agarose gel electrophoresis. The gel was visualized using a transilluminator (Baygene), and the indel efficiency was quantitatively determined using ImageJ software.

For PCSK9 base editing, Hepa1-6 cells were seeded in 24-well plates and incubated for 12-24 h to achieve 40% confluence. A mixture of 0.1 μg hyCBE mRNA and 0.05 μg sgRNA (CAGGTTCCATGGGATGCTCT) targeting PCSK9 was formulated and complexed with FD17 at an N/P ratio of 4. To this mixture, 15 μL of RNase-free water was added, followed by incubation at room temperature for 15 min. Subsequently, 35 μL of FBS-free cell culture medium was incorporated and incubated for another 15 min. The complexes were then diluted with 200 μL of FBS-free medium before replacing the media in the 24-well plates with the complex mixture. After 6 h of incubation, 500 μL of cell culture medium containing 10% FBS was added to the cells. Following 72 h of mRNA delivery, the editing efficiency was assessed Sanger sequencing.

**Cellular uptake assay:** HeLa cells or 143B cells were cultured in 24-well plates for 24 h before *in vitro* gene transfection (80-90 % confluent), then incubated with endocytosis inhibitors including polyinosinic acid (1.15 µM), chlorpromazine (20 µM), MβCD (5 mM), cytochalasin-D (5 µM), or wortmannin (200 nM) for 1 h. The cells were treated with the complexes formed by the fluoropolymers and luciferase mRNA in 10% FBS containing cell culture medium for 9 h. The EGFP expression of the treated cells were observed by fluorescent microscopy (Olympus, Janpan) and quantitatively measured by flow cytometry (BD FACSCalibur, USA).

**Gal8 recruitment assays****:** Gal8-YFP-HeLa were constructed by the reported method.^37^ Gal8-YFP-HeLa cells were treated with the complexes formed by the fluoropolymers and luciferase mRNA, and incubated for 0.5 h, 1.5 h, 3 h, and 6 h, respectively. The Gal8-YFP recruitment was observed by confocal microscopy (Leica SP5, Germany). The nuclei were stained by Hoechst 33342. Three repeats were conducted for each transfection. The average number and area of Gal8-YFP spots in each cell were quantified by the Image J, and 200 cells in each repeat was analyzed.

**Laser-induced CNV mouse model:** 6- to 8-week-old male C57BL/6J mice were used in the laser-induced CNV mouse model study (Shanghai Jesjie Laboratory Animal Co., LTD, China). According to a statement published by the Association for Research in Vision and Ophthalmology (ARVO), all tests were approved by the Eye, Ear, Nose and Throat Hospital Attached to Fudan University's Animal Ethics Committee (Animal Ethics Number: IRBEENT-20210301b). Mice were anaesthetized by intraperitoneal injection of avertin, and pupils were dilated with 1% tropicamides (Bausch & Lomb, China). The cornea was treated with ofloxacin eye ointment (Sinqi, China) to keep it moist. A coverslip was then affixed to the cornea to serve as an ophthalmoscope. Four burns of laser photocoagulation (532 nm wavelength, 50 μm spot size, 0.1 sec duration, and 120 mW intensity) was administered to retina by a slit lamp delivery system after the pupil had fully dilated. Four rounds of laser photocoagulation around the optic nerve and away from the major blood arteries in the retina were performed on each eye. Laser spots were located at 1-1.5 optic discs away from the optic nerve heads. Bruch's membrane rupture, which is a necessary condition for choroidal neovascularization, was identified as the cause of the vaporization bubble that was produced when the laser was applied. Mice observed to have intraocular hemorrhage were excluded from the study.

**Intravitreal injection:** Prior to intravitreal injection, mice were anesthetized with avertin (or the appropriate anesthetic) and an ocular surface anesthetic was applied to lessen local reflexes. Tropicamide eye drops were administered to dilate the pupil for improved intraocular visualization under the microscope. Once the pupil was fully dilated, the eyeball was gently stabilized with tweezers. The tip of the syringe was inserted into the vitreous cavity at a 60-degree angle from the corneal surface, approximately 1 mm beyond the corneal limbus, and 5 μL of the appropriate solution was injected into each eye. To prevent leakage, the needle was kept in place for 30 seconds after injection before being slowly withdrawn. Throughout the procedure, the position of the needle was carefully monitored to avoid contact with the lens and potential traumatic cataract.

**Intraocular distribution of delivery complex:** Male C57BL/6J mice aged 6-8 weeks underwent laser induction of CNV at four spots. Subsequently, 5 µL of mRNA delivery complexes, formulated with either FD17_Cy5.5_ or UD4_Cy5.5_, were intravitreally injected. After 1 hour, the eyes were harvested, fixed in 4% paraformaldehyde for 24 hours, dehydrated through a graded ethanol series, and then frozen for sectioning. Retinal fluorescent images were captured using confocal microscopy.

**Intraocular Cre mRNA delivery:** Male Ai14 mice aged 6-8 weeks underwent laser induction of CNV at four spots. Subsequently, 5 µL of FD17/Cre mRNA (N/P ratio of 4) was intravitreally injected. Five days later, the eyes were harvested, fixed in 4% paraformaldehyde for 24 hours, dehydrated through a graded ethanol series, and frozen for sectioning. The sections were permeabilized with 0.1% Triton X-100 for 15 minutes at room temperature (approximately 20-25°C), blocked with 3% BSA for 1 hour, and then incubated with primary antibody (mouse anti-RPE65, 1:50 dilution) at 37°C for 2 hours with gentle agitation. After washing three times with PBS for 5 minutes each, sections were incubated with anti-rabbit IgG 488 secondary antibody (1:200 dilution) at room temperature for 1 hour. Nuclei were labeled with Hoechst stain (1:2000 dilution) for 5 minutes. Sections were mounted with anti-fade mounting medium, and retinal fluorescent images were captured using confocal microscopy.

**In vivo treatment of CNV:** Mice were randomly divided into 6 groups: 1) Control, 2) CNV, 3) FD17/Cas9 mRNA/sgRNA complexes targeting VEGFA A (FD17/VEGFA), 4) RNA only, 5) unmodified generation 4 PAMAM dendrimer/Cas9 mRNA/sgRNA (UD4/VEGFA), and 6) FD17/Cas9 mRNA/scrambled sgRNA (FD17/NC). The mice in each group were intravitreally injected with corresponding formulation. For each eye, the concentrations of FD17, Cas9 mRNA, sgRNA, and UD4 were 2 μg, 0.3 μg, 0.15 μg, and 2 μg, respectively.

**Gene editing assay in vivo:** After treatment, the RPE cells of each mouse was extracted, and genomic DNA was extracted from the RPE cells using the FastPure Cell/Tissue DNA Isolation Mini Kit (DC102, Vazyme) as per standard protocols. The extracted DNA was then amplified with two pairs of specific PCR primers (F: CAGGCCATGTCCCTTGGAAC; R: CCAAAGATGCCCACCTGCAT) using Phanta Max Master Mix (P515, Vazyme) to generate templates for Sanger sequencing.

**FFA:** FFA images were captured within 1 minute after 0.02 ml of a 10% sodium fluorescein (Alcon) solution was injected intraperitoneally.

**OCT:** OCT images of the eyes were captured using Spectralis SD-OCT (Heidelberg, Germany). Images of hyper reflexes represented CNV regions. The direction of the long axis is in accordance with the RPE level, which was determined to be the width of the CNV lesion; the direction of the lesion's height is perpendicular to the long axis. The lesion's width and heihgt were measured with imageJ in photos scanned through the lesion's center.

**Choroidal-retinal flat mount and immunostaining:** The eyeballs were preserved in 4% paraformaldehyde (PFA) for an hour. The pigment epithelium (RPE)-choroid-sclera (RCS) complex was sliced in a four-leaf configuration to prevent curling after all extraocular tissues, including the conjunctiva, muscles, and optic nerve, had been totally removed. After three washes with 1×PBS (5 min each), the tissue was sealed for an hour with 10% goat serum (including 0.03% Triton X-100) + broken membrane. Washed the tissue three times with 1×PBS (5 min each) after incubated with isolectin IB4 (1:50) overnight at 4°C. Flattened choroidal-retinal flat mounts were positioned on a microscope slide with the vitreous side facing up and the photoreceptor layer facing down, and then covered with a cover glass. Images of flat mounts were captured with a Zeiss Aiox506 fluorescence microscope at an excitation wavelength of 488nm and a magnification of 5×. Quantitative comparison between various groups was made by measuring the relative CNV area [(CNV area) / (total choroid area)] using ImageJ.

**ERG:** Each group of mice underwent a 12-hour dark acclimation period prior to the ERG recording. After that, anesthetic was given and the mice's eyes were dilated. The ground electrode is positioned beneath the skin of the thigh and the reference electrode is implanted subcutaneously above nasal bone. A pair of 3mm gold S9 ring electrodes were attached to the cornea protected by moisturizer to perform ERG stimulation and recording. Rod and mixed cone/rod responses were elicited by progressively intensified flashes under dark adaption conditions, including five steps (0.01, 0.1, 1, 3 and 10cd *s/m2). The amplitudes of each step were provided after averaging with the Espion E3 instrument (diagnostics, Boxborough, MA).

**Frozen section and TUNEL staining:** Fixated enucleated eyes in 4% PFA overnight at 4 ℃. Dehydrated the fixated eyes with an increasing concentration of sucrose (20% to 30%). Embedded the eyes in cryomold containers with OCT compound and froze them over 6 h. Cut eyes in cryostat pieces of 12 μm. The frozen sections were rinsed three times with PBS following a 15 min fixation in 4% PFA. Sections were subjected to terminal deoxynucleotide transferase solution for 60 min at 37 ° C after being penetrated with 0.1% Triton X-100.The sections were counterstained with the nucleic acid stain DAPI (Sigma-Aldrich, D9542) and photographed using a confocal microscope with 40× oil-immersion objection lenses (TCS SP8; Leica Microsystems).

**Statistical analysis:** Data were presented as means ± SE. The statistical significance was determined using the analysis of variance and one-tailed Student’s t test. Statistical significance was noted as follows: ^n.s.^P ≥ 0.05, *P < 0.05, **P < 0.01, and ***P < 0.001.


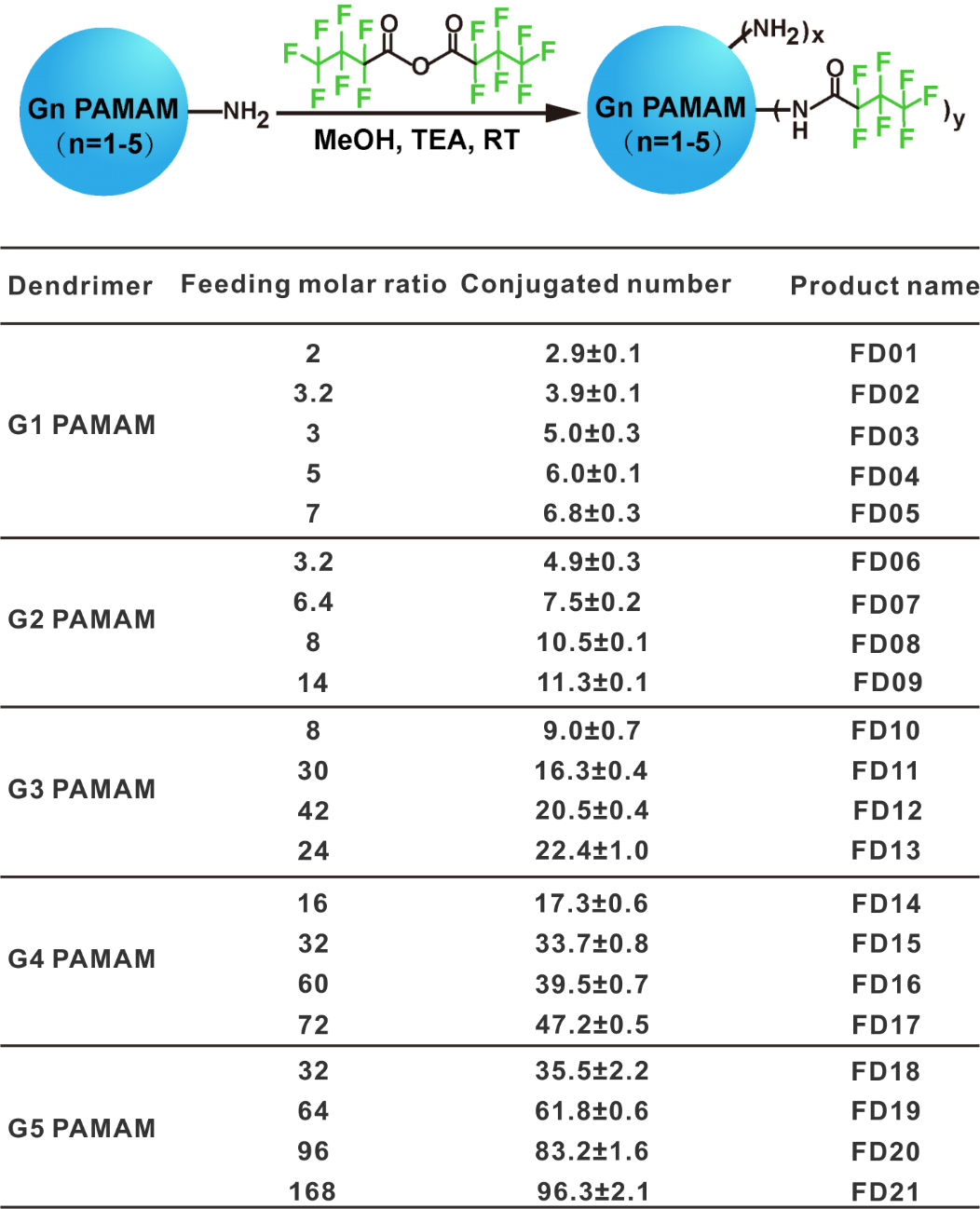


**Table S1.** Synthesis and characterization of the fluoropolymers. The feeding molar ratio means the molar ratio of the heptafluorobutyric anhydride to dendrimer during the reaction. Conjugated number means the average number of the fluorous ligands modified on each dendrimer was characterized by ninhydrin assay^1^.


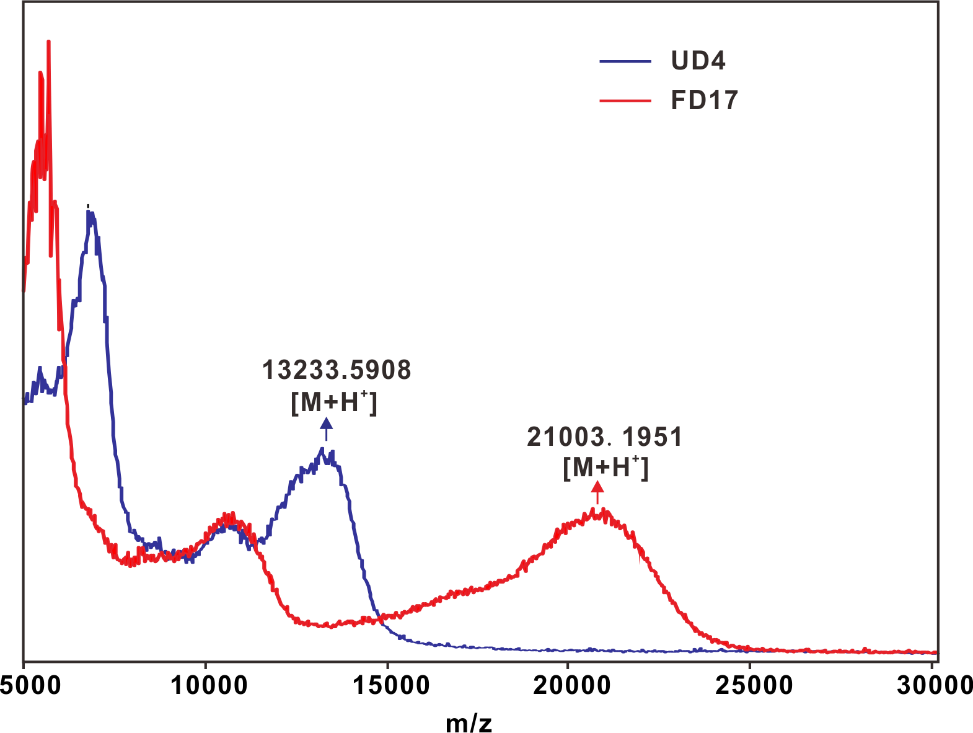


**Figure S1.** MALDI-TOF MS spectra of UD4 and FD17.


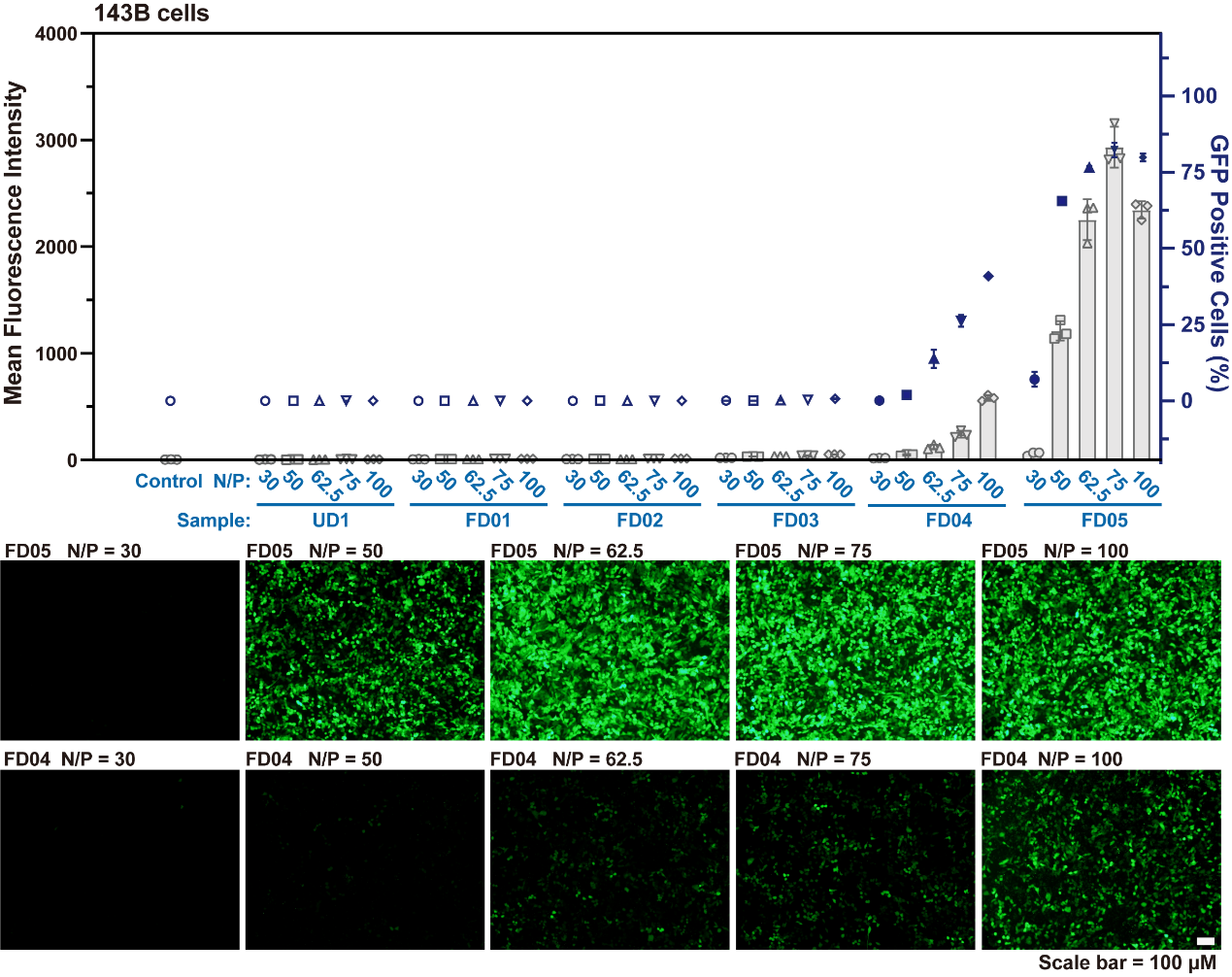


**Figure S2.** EGFP mRNA delivery efficacy of the fluorinated G1 PAMAM dendrimers in 143B cells. The mRNA delivery experiments were conducted for 24 hours, and the N/P ratios were ranged from 30:1 to 100:1.


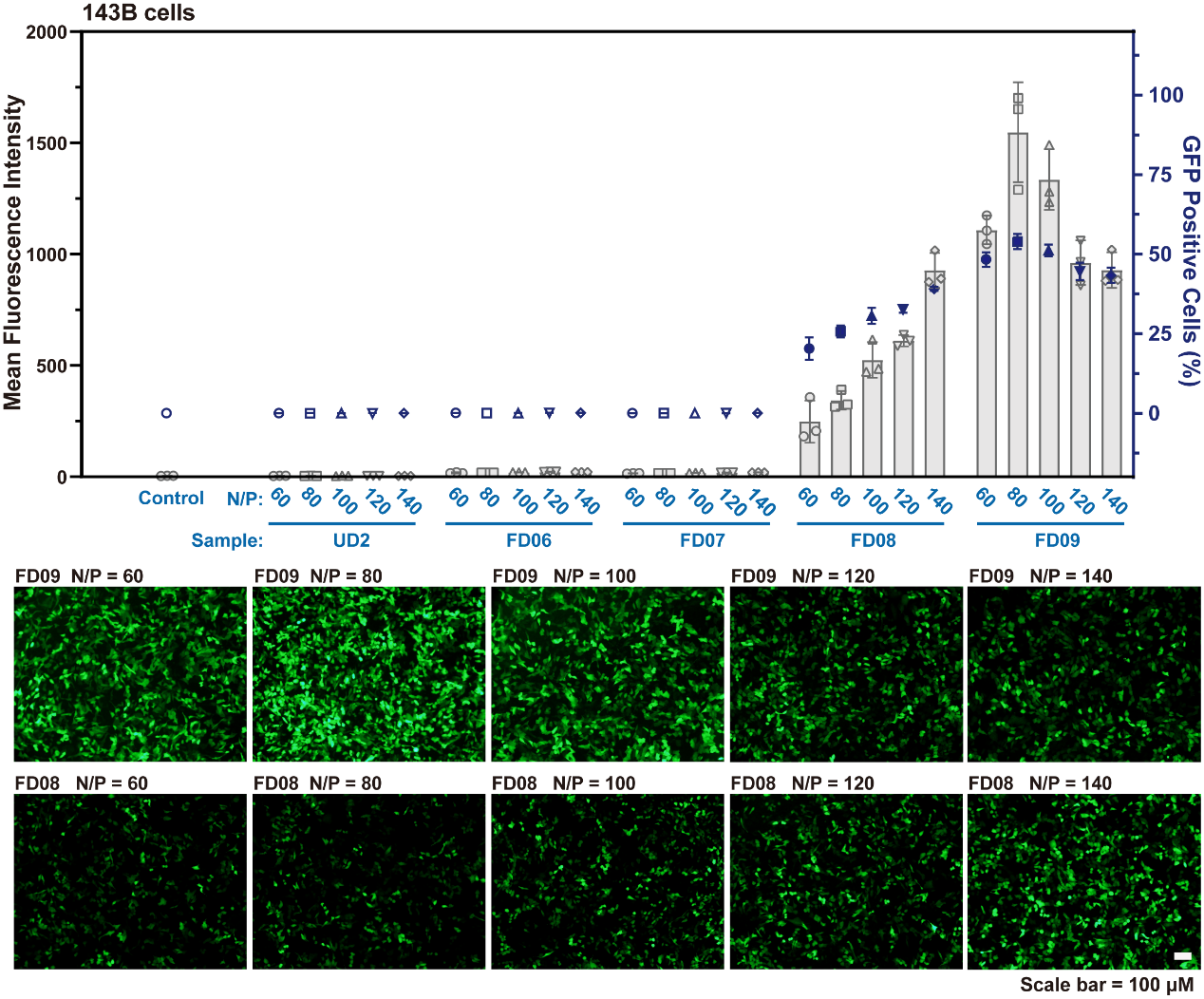


**Figure S3.** EGFP mRNA delivery efficacy of the fluorinated G2 PAMAM dendrimers in 143B cells. The mRNA delivery experiments were conducted for 24 hours, and the N/P ratios were ranged from 60:1 to 140:1.


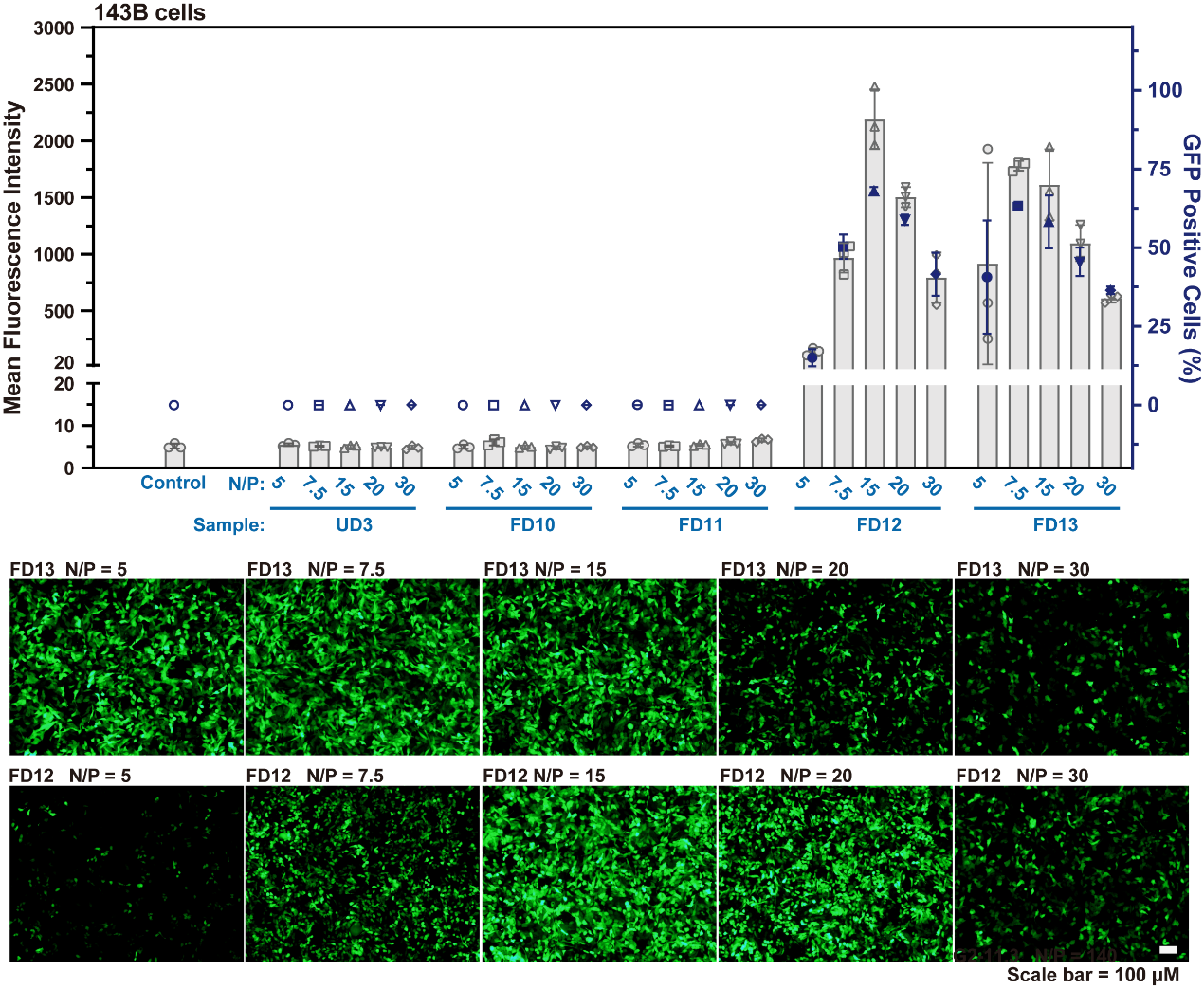


**Figure S4.** EGFP mRNA delivery efficacy of the fluorinated G3 PAMAM dendrimers in 143B cells. The mRNA delivery experiments were conducted for 24 hours, and the N/P ratios were ranged from 5:1 to 30:1.


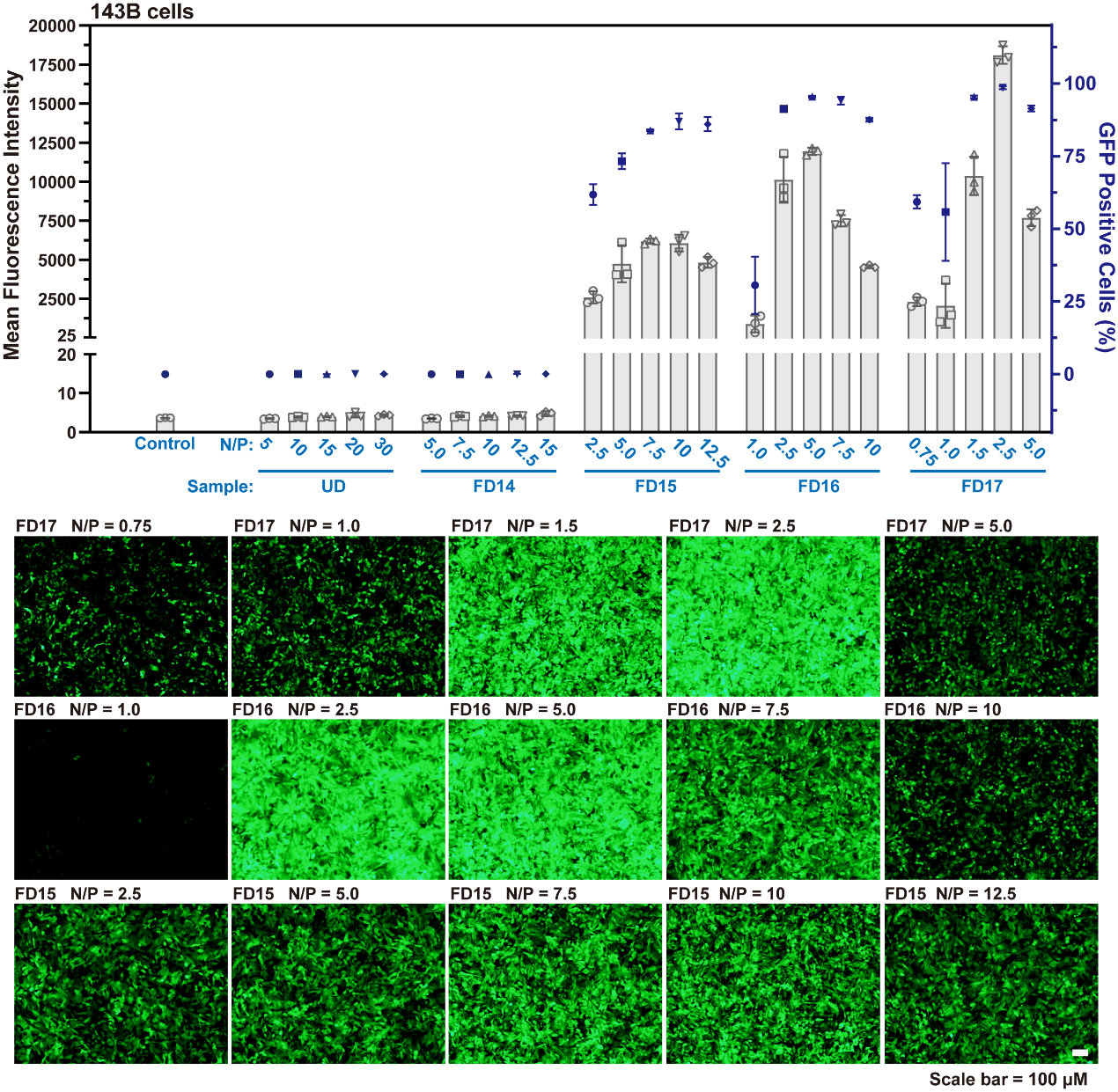


**Figure S5.** EGFP mRNA delivery efficacy of the fluorinated G4 PAMAM dendrimers in 143B cells. The mRNA delivery experiments were conducted for 24 hours, and the N/P ratios were ranged from 0.75:1 to 30:1.


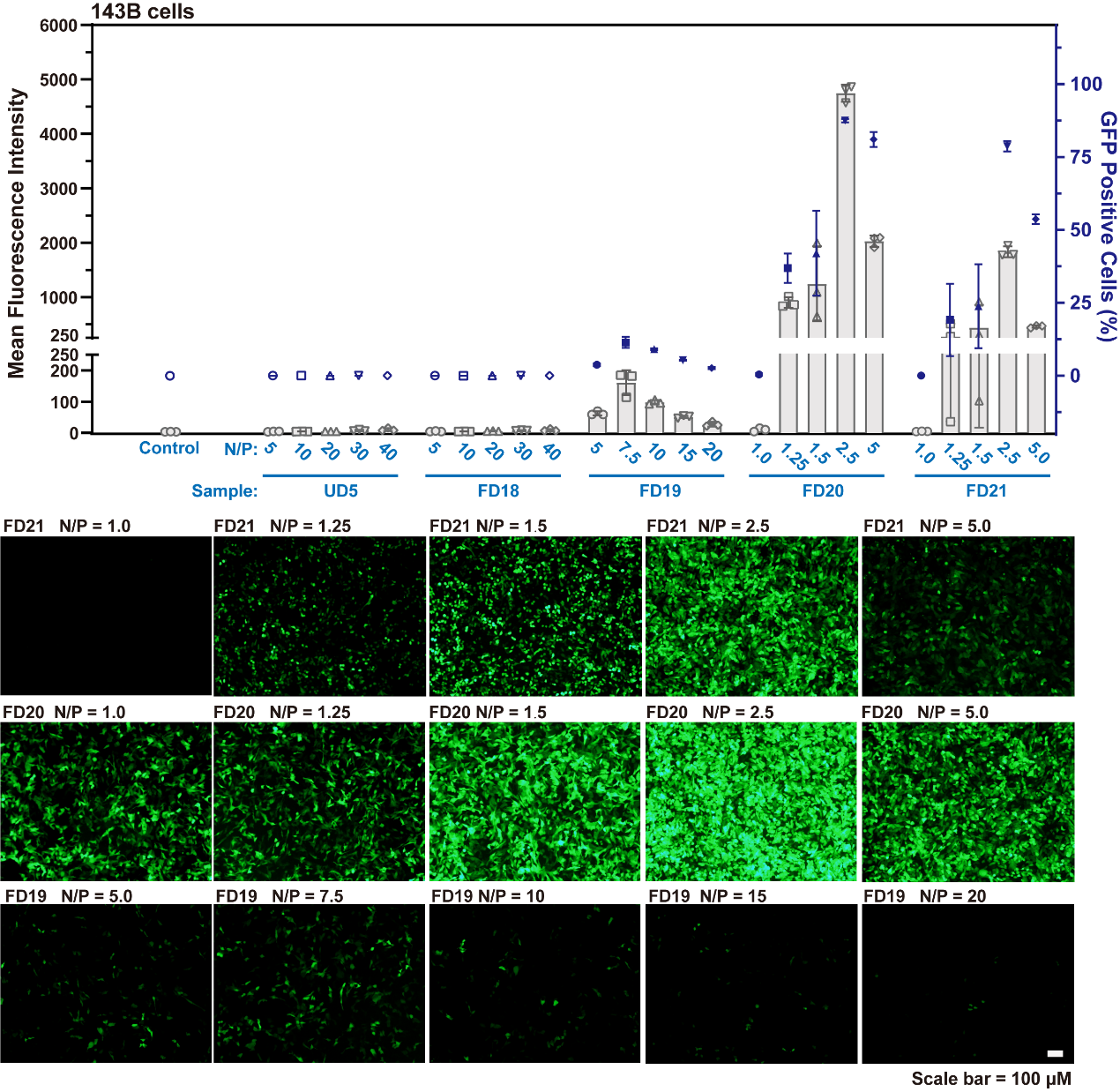


**Figure S6.** EGFP mRNA delivery efficacy of the fluorinated G5 PAMAM dendrimers in 143B cells. The mRNA delivery experiments were conducted for 24 hours, and the N/P ratios were ranged from 1:1 to 40:1.


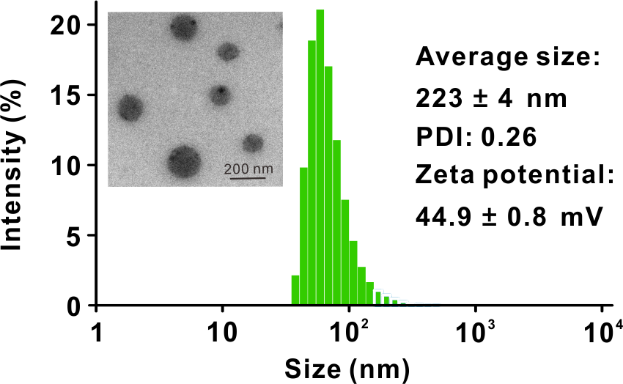


**Figure S7.** Size distribution, zeta potential, and TEM image of FD17/GFP mRNA complexes prepared at an N/P ratio of 2.5.


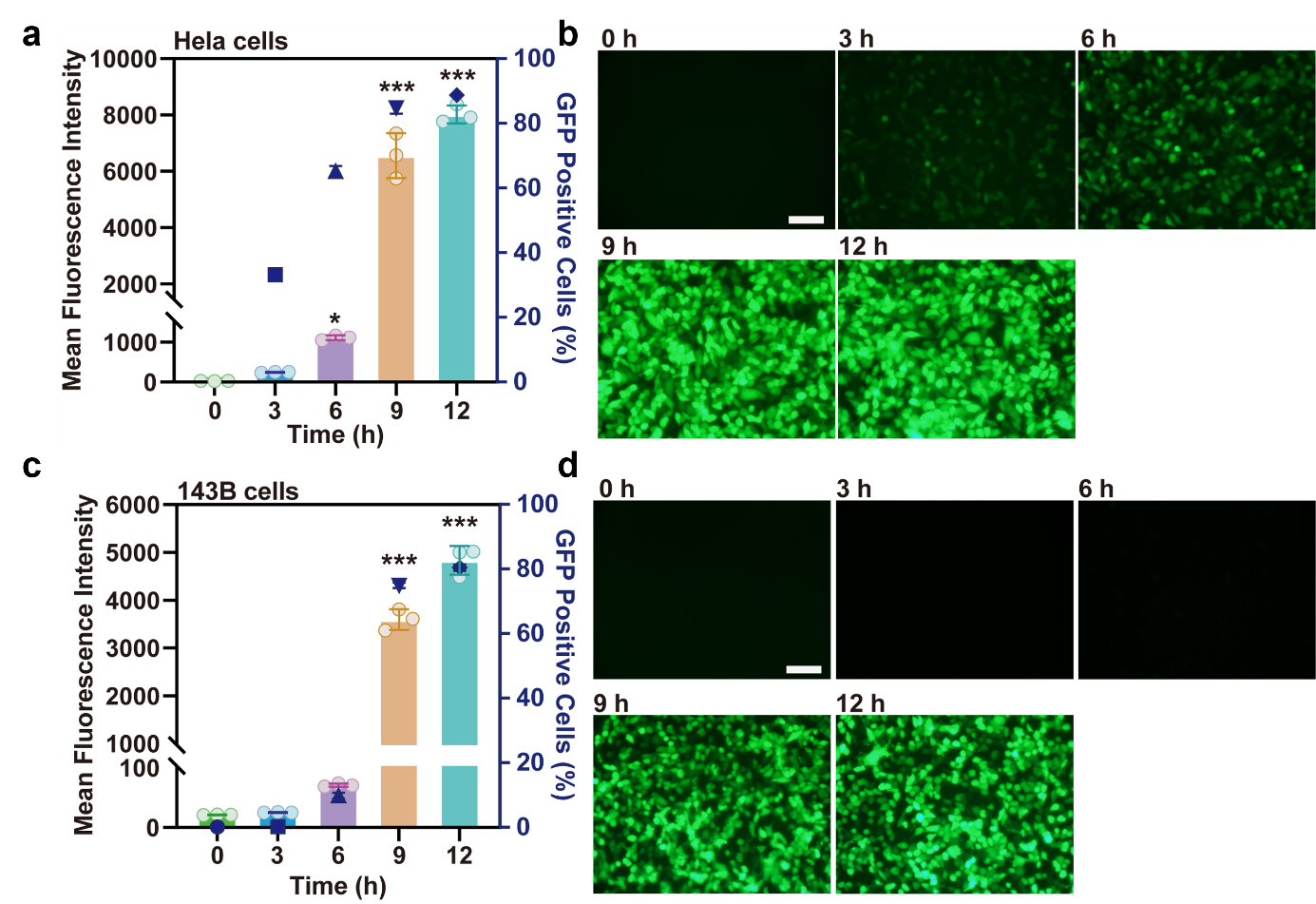


**Figure S8.** Time-dependent EGFP mRNA delivery efficacy of FD17 on HeLa and 143B cells. The flow cytometry analysis of HeLa (a) and 143B (c) cells after treated with the FD17/EGFP mRNA complexes for 0-12 h. The fluorescence images of HeLa (b) and 143B (d) cells after treated with the FD17/EGFP mRNA complexes for 0-12 h. The concentration of EGFP mRNA was 0.8 μg/mL, and the N/P ratio of the FD17 to mRNA complex was 2.5. The scale bar is 100 μm.


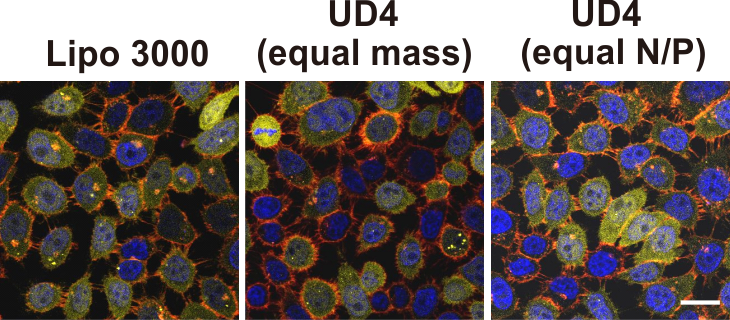


**Figure S9.** Fluorescent microscopy images of Gal8-YFP-HeLa cells exposed to Lipo 3000/mRNA, UD4/mRNA, and FD17/mRNA complexes at 6 hours. The UD4 and FD17 at equal mass or equal N/P ratio. The scale bar is 20 μm.


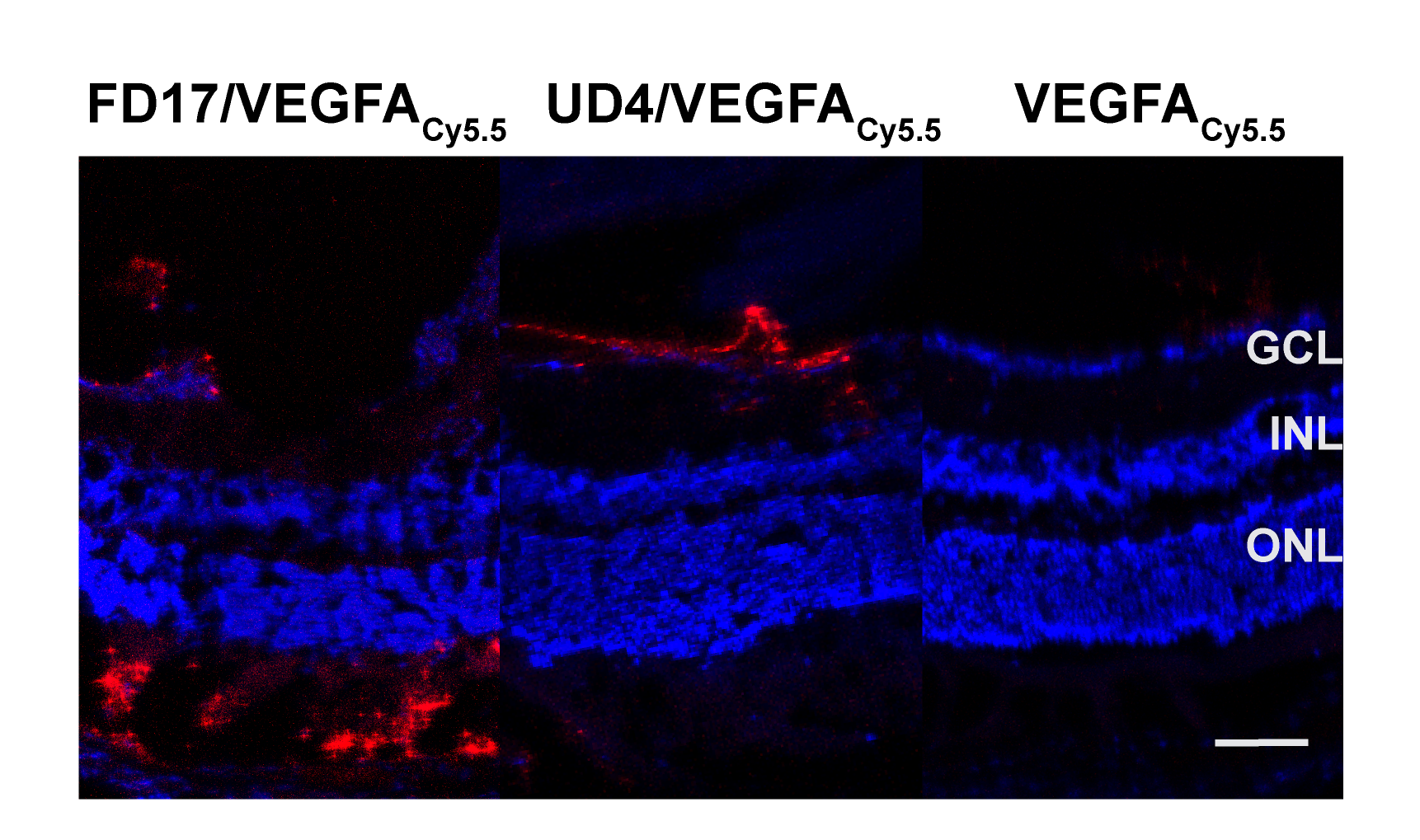


**Figure S10.** Confocal microscopy photographs show the distribution of the FD17/Cas9 mRNA/sgRNA_Cy5.5_ (FD17/VEGFA_Cy5.5_), UD4/Cas9 mRNA/sgRNA_Cy5.5_ (UD4/VEGFA_Cy5.5_) complexes, and Cas9 mRNA/sgRNA_Cy5.5_ (VEGFA_Cy5.5_) 1 hour after intravitreal injection. The cell nucleus is stained with DAPI. The sgRNA was labelled with Cy5.5. The scale bar is 75 μm.


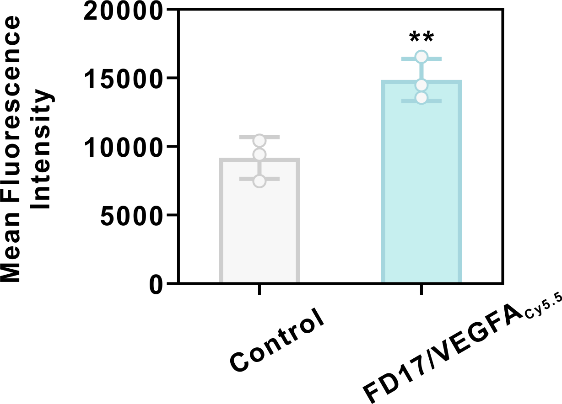


**Figure S11.** Mean fluorescence intensity of Cy5.5 in the blood of mice after 1-hour intravitreal injection of FD17/VEGFA_Cy5.5_ complexes. Mice injected with PBS were set as the control group.


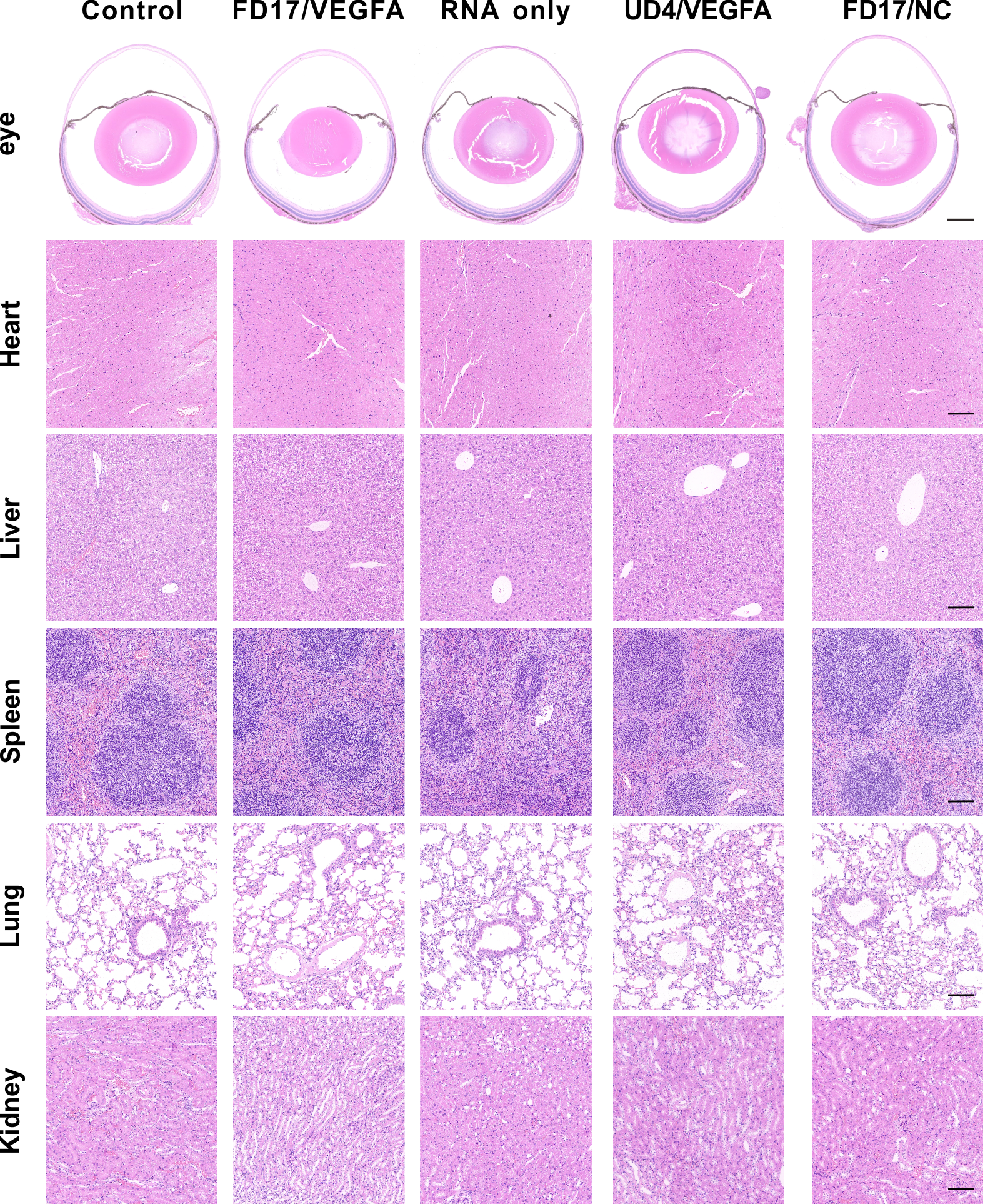


**Figure S12.** H&E staining slices of eye (Scale bar: 500 μm), heart, liver, spleen, lung and kidney (Scale bar: 100 μm) collected from mice in different groups.

**References**

1. Wang, M.; Liu, H.; Li, L.; Cheng, Y. A Fluorinated Dendrimer Achieves Excellent Gene Transfection Efficacy at Extremely Low Nitrogen to Phosphorus Ratios. *Nat. Commun.* **2014**, *5* (1), 3053.
